# Grabbing the Bull by Both Horns: Bovine Ultralong CDR-H3 Paratopes Enable Engineering of ‘Almost Natural’ Common Light Chain Bispecific Antibodies Suitable For Effector Cell Redirection

**DOI:** 10.3389/fimmu.2021.801368

**Published:** 2022-01-11

**Authors:** Daniel Klewinghaus, Lukas Pekar, Paul Arras, Simon Krah, Bernhard Valldorf, Harald Kolmar, Stefan Zielonka

**Affiliations:** ^1^ Protein Engineering and Antibody Technologies, Merck Healthcare KGaA, Darmstadt, Germany; ^2^ Chemical and Pharmaceutical Development, Merck KGaA, Darmstadt, Germany; ^3^ Institute for Organic Chemistry and Biochemistry, Technische Universität Darmstadt, Darmstadt, Germany

**Keywords:** bovine ultralong CDR-H3 antibodies, bispecific antibodies, effector cell redirection, NK cell engagers, common light chain, antibody engineering, yeast surface display

## Abstract

A subset of antibodies found in cattle comprises ultralong CDR-H3 regions of up to 70 amino acids. Interestingly, this type of immunoglobulin usually pairs with the single germline VL gene, V30 that is typically very conserved in sequence. In this work, we have engineered ultralong CDR-H3 common light chain bispecific antibodies targeting Epidermal Growth Factor Receptor (EGFR) on tumor cells as well as Natural Cytotoxicity Receptor NKp30 on Natural Killer (NK) cells. Antigen-specific common light chain antibodies were isolated by yeast surface display by means of pairing CDR-H3 diversities following immunization with a single V30 light chain. After selection, EGFR-targeting paratopes as well as NKp30-specific binders were combined into common light chain bispecific antibodies by exploiting the strand-exchange engineered domain (SEED) technology for heavy chain heterodimerization. Biochemical characterization of resulting bispecifics revealed highly specific binding to the respective antigens as well as simultaneous binding to both targets. Most importantly, engineered cattle-derived bispecific common light chain molecules elicited potent NK cell redirection and consequently tumor cell lysis of EGFR-overexpressing cells as well as robust release of proinflammatory cytokine interferon-γ. Taken together, this data is giving clear evidence that bovine bispecific ultralong CDR-H3 common light chain antibodies are versatile for biotechnological applications.

## Introduction

The human body is continuously exposed to potentially life-threatening opponents such as bacteria, viruses or cancerous cells. In order to assert oneself, antibodies (Abs) play a fundamental role in host defense by recognizing foreign antigen in an adaptive fashion. The high specificity for a given antigen in conjunction with humoral and cellular effector functions mediated by the Fc-part of IgG isotypes renders this class of Abs as very promising molecules for therapy (1, 2). This is exemplified by the fact that as of 2021 around 100 therapeutic antibody derivatives have been granted marketing approval by the FDA (3). However, one obvious obstacle of monoclonal antibodies for therapeutic purposes results from their monospecific nature since diseases are typically multifaceted e.g. with respect to their origin or disease mediators (4, 5). Consequently, tremendous efforts were made within the last decades to engineer antibodies for bi- and multispecificity (6), culminating in the approval of four bispecific entities until now (7, 8) - including Catumaxomab that has been withdrawn in 2017 (9). Moreover, a steady incline in investigational bispecifics that are entering clinical development on a yearly basis can be observed (9, 10). Most of the molecules that are currently investigated in clinical trials are so called asymmetric formats (9). This type of bispecific antibody (bsAb) resembles the IgG-like architecture of conventional monoclonal antibodies as closely as possible. Here, each Fab arm targets a different antigen in a monovalent manner. Consequently, two different heavy chains as well as two separate light chains need to be expressed and even more importantly, assembled precisely to represent a functional bsAb. To facilitate heavy chain heterodimerization as well as specific heavy and light chain pairing, several different technologies have been developed (11). In this respect, the issue of accurate heavy and light chain assembly can be obviated by engineering common light chain bsAbs i.e. bispecifics where both Fabs share the identical light chain (12–14). Besides, an unprecedented multitude of different bsAb formats has been engineered (15), including bi- and multispecifics derived from camelids (16–18) or sharks (19–21).

**Figure 1:**
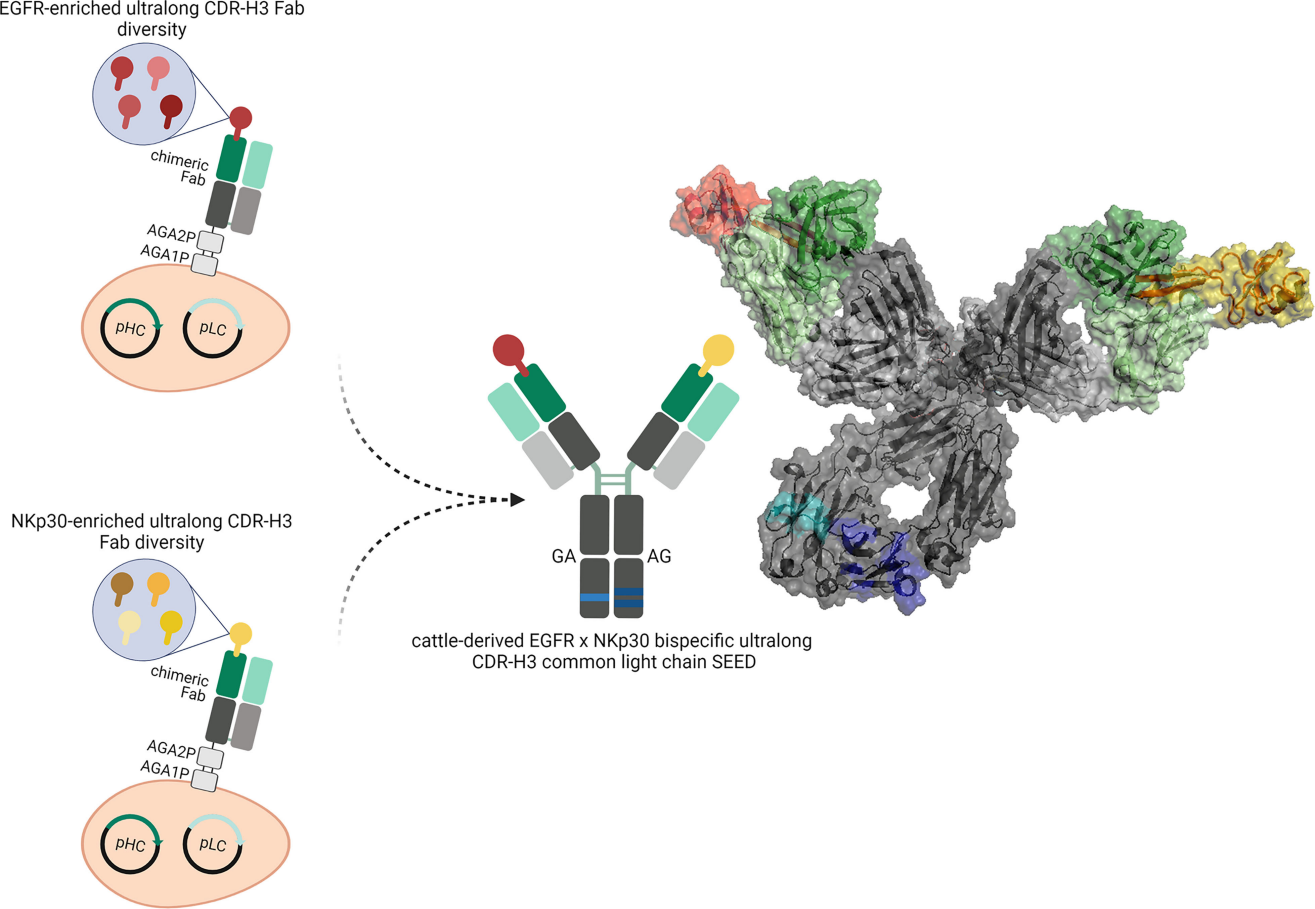
Overview about the generation of cattle-derived ultralong CDR-H3 common light chain bispecific antibodies. After immunization of cattle and library generation antigen-specific paratopes are enriched against both targets (shown in red color and yellow color). To this end, ultralong CDR-H3 regions encoding for stalk/knob architectures are specifically amplified and grafted onto a fixed chimeric Fab scaffold utilizing a single light chain. After selection, common light chain paratopes are reformatted into an IgG-like bispecific format exploiting a heavy chain heterodimerization technique (e.g. the SEED technology). Schemes generated using biorender (www.biorender.com). Model constructed with PYMOL v0.99 based on pdb entries 5dk3 and 5ilt. Individual paratopes based on stalk/knob structures are colored in red and yellow, respectively. Fixed VH region based on IGHV1-7 shown in dark green, utilized VL30 exploited as common light chain shown in light green. Constant regions of the heavy chains colored in dark grey, CLλ shown in light grey. Use of heavy chain heterodimerization technology resulting in two distinct heavy chains indicated by the use of dark blue and light blue segments.

A fraction of about 10% of the immunoglobulin repertoire found in cattle produces antibodies with exceptionally long CDR-H3 regions of up to 70 amino acids (22). Typically, the vast majority of clones harboring ultralong paratopes adopts a characteristic structure that can be divided into a stalk region composed of an ascending as well as a descending β-strand and a disulfide-rich globular architecture referred to as knob (**Figure 1**) (23). Usually, one distinct V gene segment, IGHV1-7 is utilized for the construction of bovine ultralong CDR-H3 antibodies as well as one particular germline D segment, IGHD8-2, encoding for the stalk-knob structure. IGHD8-2 is diversified in a process involving cytidine deaminase with a strong bias towards the introduction of cysteine residues causing extraordinary structural diversity through the formation of different disulfide bond patterns predominantly in the knob region (22, 24). Consequently, it is the knob region that plays a pivotal role for antigen binding, whereas the stalk as well as the VH scaffold seem to have a stabilizing function (22, 23). Intriguingly, ultralong CDR-H3 heavy chains typically pair with a single VL gene, VL30 that generally is relatively sequence conserved (25). In this respect, several of the published crystal structures of ultralong CDR-H3 antibodies share a CDR-identical light chain (24, 26, 27). Hence, these molecules comprise an almost natural source of common light chain antibodies.

In this work, we have engineered EGFR and NKp30 targeting cattle-derived bispecific common light chain antibodies that can be utilized to efficiently redirect NK cells in order to kill EGFR-overexpressing tumor cells. EGFR is a receptor tyrosine kinase overexpressed in an array of different tumors (28–30). We have recently described the generation of a platform process for isolating ultralong CDR-H3 antibodies targeting EGFR by combining cattle immunization with yeast surface display (31). To this end, bovine ultralong CDR-H3 regions were PCR-amplified and grafted onto a fixed IGHV1-7 scaffold. Subsequently, this CDR-H3-only diversity was combined with a single VL30 light chain enabling the facile isolation of EGFR-specific antibodies. In this work, we have isolated multiple NKp30-specific ultralong CDR-H3 antibodies by exploiting the same platform process involving the identical VL30 light chain. NKp30 is an activating NK cell receptor that can be addressed in a bispecific fashion to efficiently trigger NK-cell mediated target cell lysis (32–34). Following isolation of NKp30-specific paratopes by yeast surface display (35, 36), NKp30-addressing clones as well as EGFR targeting variants both sharing the identical light chain were combined into common light chain bispecifics by employing the strand-exchange engineered domain (SEED) technology for heavy chain heterodimerization (**Figure 1**) (37). The vast majority of resulting common light chain bsAbs displayed favorable biophysical properties as well as simultaneous binding to both antigens in the nanomolar range. Most importantly, generated IgG-like bsAbs facilitated significant NK cell-mediated lysis of EGFR-overexpressing A431 tumor cells as well as a robust release of proinflammatory cytokine interferon-γ (IFN-γ). Taken together, our data demonstrates that cattle-derived bispecific common light chain ultralong CDR-H3 antibodies can be readily engineered that seem to be versatile for biomedical applications such as effector cell redirection.

## Material and Methods

### Immunization

As previously described, three cattle (*Bos taurus*) with approximately one year of age were immunized using recombinant human NKp30 extracellular domain comprising a *C*-terminal hexahistidine tag (ECD; produced in-house) in a cocktail approach with recombinant *C-*terminal his-tagged EGFR ECD (produced in-house) at preclinics GmbH, Germany (31). Animal care and invasive procedures were in accordance with local animal welfare protection laws and regulation (Niedersächsisches Landesamt für Verbraucherschutz und Lebensmittelsicherheit (LAVES), Dezernat 33 – Tierschutzdienst. Number: 33.19-42502-05-17A210). Six immunizations were performed in total over the period of 84 days (d0, d28, d42, d56, d70, d84). To this end, 200 µg of NKp30 (in a volume of 2 ml) were mixed with 2 ml Fama adjuvant (GERBU Biotechnik) and injected subcutaneously at multiple sites. After immunization (day 88) 250 ml of blood per specimen was collected followed by RNA extraction and cDNA synthesis.

### Yeast Surface Display Library Generation

The library construction process involving strains, reagents as well as plasmids has been described in detail elsewhere (31). In brief, *S. cerevisiae* strain EBY100 *MATa (URA3-52 trp1 leu2Δ1 his3Δ200 pep4::HIS3 prb1Δ1.6R can1 GAL (pIU211:URA3*)) was utilized for the generation of the heavy chain diversity, whereas BJ5464 cells (*MATα URA3-52 trp1 leu2Δ1his3Δ200 pep4::HIS3 prb1Δ1.6R can1 GAL*) harboring the light chain plasmid (pLC) encoding for a specific VLλ30 (**Supplementary Figure S1**) was exploited. Primer sets for specific ultralong CDR-H3 amplification are given in **Supplementary Table 1**. For PCR-based amplification 1 µl of cDNA pooled from all three specimen was used in a final volume of 50 µl as well as Q5 High-Fidelity 2x Master Mix (New England Biolabs; NEB). Conditions were as followed: 98°C for 3 min, 35 cycles of 30 s at 98°C and 50 s at 72°C, followed by 2 min at 72°C. PCR products were purified by Wizard^®^ SV Gel and PCR Clean-up System (Promega). Gap repair cloning was employed for library construction according to Benatuil and co-workers (38). Therefore, 12 µg CDR-H3 PCR product as well as 3.5 µg *Not*I and *Eco*RI (both New England Biolabs) digested heavy chain destination plasmid (pHC) were used per electroporation reaction. The resulting library size was roughly estimated by dilution plating on SD-Trp agar plates. In order to accomplish Fab display, EBY100 cells comprising the heavy chain diversity as well as BJ5464 cells harboring the single light chain were combined by yeast mating (39, 40).

### Selection of NKp30-Targeting Ultralong CDR-H3 Fabs

Library sorting was facilitated by growing diploid library cells overnight in SD-Trp-Leu medium at 30°C and 120 rpm agitation. Subsequently, library cells were transferred to SG-Trp-Leu medium supplemented with 10% (w/v) polyethylene glycol 8000 at an OD_600_ of 1.0 and incubated for 2 days at 20°C and 120 rpm. Afterwards, cells were washed twice with PBS (Sigma Aldrich) and incubated with *C*-terminally hexahistidine tagged recombinant human NKp30 ECD (produced in-house or Abcam) at a concentration of 1 µM for 30 min on ice. Cells were washed thrice, followed by simultaneous detection of functional Fab display and antigen binding. To this end, cells were labeled with light chain specific goat F(ab’)2 anti-human lambda R-phycoerythrin (R-PE) (SouthernBiotech, diluted 1:20) as well as Penta-His Alexa Fluor 647 Conjugate antibody (Qiagen, diluted 1:20) for sorting round one or SureLight^®^ APC Anti-6X His tag^®^ antibody (abcam, diluted 1:20) for sorting round two. Eventually, library cells were washed thrice with PBS and selected by fluorescence-activated cell sorting (FACS) on a BD FACSAria™ Fusion cell sorter (BD Biosciences). In the first round of selection, a total number of approx. 5 x 10^8^ cells were sorted to ensure adequate coverage of the library. For the second round of library sorting about 5 x 10^7^ cells were exploited.

### SEEDbody Expression and Purification

Monovalent SEED antibody derivatives of NKp30-targeting cattle-derived ultralong CDR-H3 paratopes as well as bispecific common light chain SEEDbodies were designed in-house, synthesized and subcloned into pTT5 vector backbone by GeneArt (Thermo Fisher Scientific). Therefore, NKp30-specific VH regions were placed onto the AG chain of the SEEDbody encoding for human constant regions. EGFR-targeting VH domains were grafted onto the GA chain, also encoding for human constant regions. In both heavy chains we implemented amino acid mutations L234A, L235A, P329G to abolish Fc-mediated immune effector functions (41). The bovine VLλ30 region was fused to human CLλ. For monovalent (one-armed) SEEDbody expression of NKp30-addressing cattle derived entities, respective AG chain plasmids were co-transfected with the light chain plasmid as well as a paratope-less GA chain plasmid (i.e. the GA chain starting from the hinge region) in a 2:1:1 (AG:GA:LC) ratio. For bsAb expression, AG chain plasmids encoding for NKp30 paratopes were combined with GA chain plasmids encoding for EGFR-specific cattle-derived common light chain paratopes as well as with the light chain plasmid in a 2:1:1 (AG:GA:LC) ratio. In general, 25 ml Expi293™ cells were transfected with the respective expression vector mixtures according to the manufacturer’s recommendations and protocols (Thermo Fisher Scientific). Supernatants were collected after five days and purified using MabSelect chromatography resin (GE healthcare). Subsequently, buffer was exchanged to PBS pH 6.8 *via* Pur-A-Lyzer™ Maxi 3500 Dialysis Kit (Sigma Aldrich/Merck KGaA) for 24 h at 4°C. Optionally, in case of low yields, a concentration step was executed using Amicon Ultra-4 Centrifugal Filters (MW cutoff 10 kDa, EMD Millipore). Protein concentrations were determined on the QIAexpert system (Qiagen). Analytical size exclusion chromatography was exploited to determine aggregation propensities using a TSKgel SuperSW3000 column (4.6 × 300 mm, Tosoh Bioscience LLC) in an Agilent HPLC system with a flow rate of 0.35 ml/min.

### Biolayer Interferometry

All BLI measurements were performed on the Octet RED96 instrument (ForteBio, Pall Life Science) at 25°C and 1000 rpm, agitation. To assess binding as well as for kinetic measurements, cattle derived bsAbs were loaded onto anti-human Fc (AHC) sensors at a concentration of 5 µg/ml (in PBS) for 3 min followed by 60 s of sensor rinsing using kinetics buffer (KB; PBS + 0.1% (v/v) Tween-20 + 1% (w/v) BSA). Subsequently, association to the respective antigen was measured at varying concentrations (100 nM, 50 nM, 25 nM and 12.5 nM for EGFR and depending on the bsAb at 100 nM, 50 nM, 25 nM, 12.5 nM, 6.25 nM and 3.125 nM for NKp30) for 300 s followed by dissociation in KB for 300 s. For analyzing simultaneous binding on the protein level, bsAbs were loaded onto AHC sensors at a concentration of 5 µg/ml (in PBS) for 3 min followed by 60 s of sensor rinsing in KB. Afterwards, a first association step was performed using 100 nM NKp30 (Abcam) for 200 s followed by a second association in EGFR at 100 nM for 200 s. To perform competition assays with B7-H6, bsAbs were loaded onto AHC sensors at a concentration of 5 µg/ml (in PBS) for 3 min followed by 60 s of sensor rinsing in KB. A first association was performed using NKp30 at 100 nM for 100 s followed either by 100 s in KB or 100 s in 1000 nM B7-H6 ECD. Data was fitted (1:1 binding model) and analyzed using ForteBio data analysis software 8.0 as well as Savitzky-Golay filtering.

### Flow Cytometry

Cellular binding was assessed on a Sartorius iQue3 flow Q1 cytometer and the IntelliCyt ForeCyt software was used for analysis. For each experiment, 800-1800 cells per well were measured. To this end, 10^5^ cells/well were seeded and incubated for 1 h on ice with bsAbs at 100 nM in PBS supplemented with 1% (w/v) BSA after two initial washing steps with PBS+1 % (w/v) BSA. Following antibody incubation, two additional washing steps with PBS+1% (w/v) BSA were performed with subsequent Alexa Fluor^®^ 488 AffiniPure Fab Fragment Goat Anti-Human IgG (Fc specific) (Jackson ImmunoResearch) detection antibody staining (200 nM) at 4°C for another 30 min. After two washing steps with PBS+1% (w/v) BSA, 20 μg/ml propidium iodide (Invitrogen) was used to label dead cells in a total volume of 100 μl/well. Controls were included, e.g. anti-HEL IgG, cells without antibody incubation as well as cells labeled with the detection reagent only. For the detection of simultaneous binding, A431 cells were seeded and labeled equivalently with bsAbs. Following bsAb incubation at a concentration of 100 nM and two washing steps, his tagged NKp30 (ECD, Acro Biosystems) was added at 200 nM for 30 min. After two additional washing steps, cells were incubated with 400 nM of detection antibody (Penta His Alexa Fluor^®^ 488 Conjugate (Qiagen)) for 30 min and 20 μg/ml propidium iodide (Invitrogen). Controls were included, e.g. anti-HEL IgG, cells without antibody incubation, cells labeled with the detection reagent only as well as cells treated with a bispecific and detection antibody, but not with NKp30.

### Killing Assay

The killing assay has been described in detail elsewhere (18). In brief, PBMCs were isolated from blood of healthy donors by density gradient centrifugation. NK cells were enriched using the EasySep™ Human NK Cell Isolation Kit (Stemcell Technologies). After overnight incubation in complete medium using low dose recombinant human IL-2 (100 U/ml, R&D systems), cells were adjusted to 0.625 x 10^6^ vc/ml. EGFR positive A431 cells or EGFR negative ExpiCHO™ cells were stained with CellTracker™ Deep Red Dye (ThermoFisher). Target cells were seeded into 384-well clear bottom microtiter plates (Greiner Bio-One) at 2500 cells/well in 20 µl volume and incubated for 3 h. Afterwards, NK cells were added at different E:T ratios (i.e. 1:1, 5:1, 10:1 and 20:1). BsAbs were added at concentrations as indicated. An EGFR targeting Fc immune effector silenced antibody derivative was utilized as negative control. SYTOX™ Green Dead Cell Stain (Invitrogen, 0.03 µM) was dispensed to the assay followed by plate incubation and on-line measurement for 24 h in the Incucyte^®^ system. Lysis was normalized to maximum lysis triggered by therapeutic antibody cetuximab or to target cells cultivated with 30 µM staurosporine (Merck Millipore). Overlay signals allowed for analysis of dead target cells only.

### Cytokine Release Assay

The EasySep™ Human NK Cell Isolation Kit (Stemcell Technologies) was employed to isolate NK cells derived from PBMCs of healthy human donors. Cell were incubated overnight in complete medium supplemented with 100 U/ml recombinant human interleukin-2 (R&D Systems). Subsequently, 2.500 A431 cells were seeded in 384 well plates. After 3 h of incubation, NK cells were added at an E:T ration of 5:1 followed by the addition of cattle-derived bsAbs at a final concentration of 50 nM. An EGFR targeting Fc immune effector silenced antibody derivative was utilized as negative control. After 24 h incubation supernatants were collected and analyzed utilizing the human IFN-γ HTRF kit (Cisbio) by following the manufacturer’s instructions. Plates were measured with PHERAstar FSX (BMG Labtech) and data were analyzed by MARS software (v.3.32, BMG) enabling a 4-parameter logistic (4PL 1/y²) model fitting of the standard curve.

## Results

### Isolation of Chimeric NKp30-Targeting Ultralong CDR-H3 Fab Fragments

**Figure 2:**
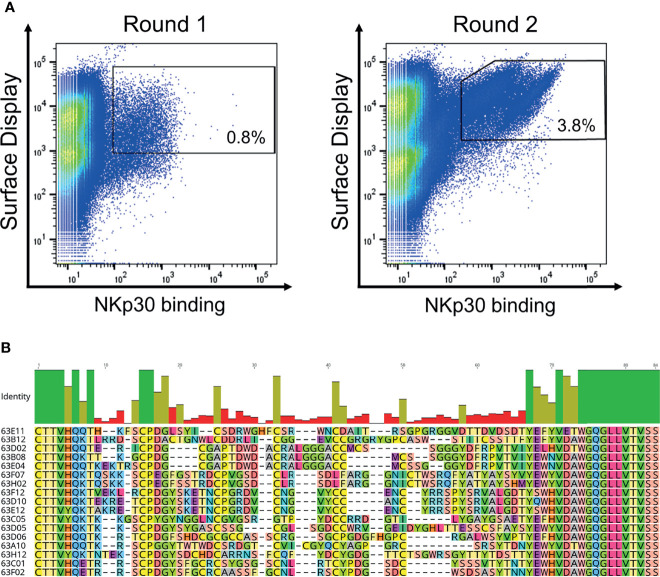
Yeast surface display based selection of NKp30 targeting chimeric bovine x human Fab fragments by yeast surface display as well as sequence analysis after enrichment. **(A)** Within two sorting rounds a NKp30-binding population was enriched. A two-dimensional sorting strategy was applied to label for functional Fab assembly as well as for NKp30 binding. To this end, library cells were incubated with recombinant human his-tagged NKp30 at a concentration of 1 µM followed by staining using secondary detection reagents directed against the his-tag as well as against the constant region of the human lambda chain. **(B)** CDR-H3 alignment of sequence unique ultralong CDR-H3 paratopes obtained after library sorting. Sequence of IGHJ2-4 is also shown. Amino acids given in 1-letter code and in different colors. Alignment generated with Geneious Prime^®^ v2021.1.1.

We have previously described the generation of a platform process for the isolation of ultralong CDR-H3 antibodies by combining cattle immunization and yeast surface display (31). The same strategy involving the same library was applied in this study for the isolation of NKp30-specific antibodies. In brief, as already described earlier (31) we specifically amplified ultralong CDR-H3 regions from cDNA obtained from the peripheral blood mononuclear cell (PBMC) repertoire of cattle that were immunized with recombinant human NKp30 ECD. Subsequently, a heavy chain library was constructed by grafting the amplified CDR-H3 diversity onto a fixed bovine IGHV1-7 scaffold fused to human domain CH1 and AGA2P by gap repair cloning into *S. cerevisiae* strain EBY100. The resulting library with approximately 5 x 10^7^ unique clones was then combined by yeast mating with BJ5464 cells harboring a single light chain plasmid encoding for a bovine V30 paratope fused to a human CLλ region (39, 40). Afterwards, the resulting diploid yeast cell Fab library was screened by fluorescence activated cell sorting (FACS) to isolate ultralong CDR-H3 common light chain paratopes specific to NKp30. To this end, a two-dimensional labeling strategy was applied to simultaneously select for full-length Fab display in addition to NKp30 binding (**Figure 2A**). Using an antigen concentration of 1 µM for selection, we were able to enrich for a NKp30-targeting population within two rounds of FACS. Sequencing of 192 clones of the sorting output revealed the isolation of 17 unique CDR-H3 paratopes on the protein level with a length ranging from 56 to 66 residues and an even number of four to eight Cys residues within that region (**Figure 2B**). Since the isolated paratopes have to be functional in a strictly monovalent fashion when reformatted into common light chain bsAbs, we initially produced all 17 cattle-derived Abs in a one-armed SEED format (**Supplementary Figure 2**). For this, we exploited the SEED technology which relies on beta-strand exchanges of IgG and IgA CH3 constant domains, preferably resulting in heavy chain heterodimerization (37). The bovine x human chimeric ultralong CDR-H3 Fab fragments were genetically fused to the AG chain of the SEED molecule, while for generating monovalent versions the GA Fc chain was expressed without paratope. For all the molecules in this study we introduced amino acid exchanges L234A, L235A, P329G into both heavy chains (41) to abolish Fc-mediated immune effector functions. After expression and protein A purification we analyzed binding to recombinant human NKp30 ECD in a biolayer interferometry (BLI) experiment using an antigen concentration of 100 nM (data not shown). This revealed a total number of 13 NKp30-specific monovalent bovine x human ultralong CDR-H3 antibody derivatives (unfunctional: 63E04, 63C05, 63F02 and 63H12).

### Generation, Biophysical, and Biochemical Characterization of Bispecific Bovine x Human Chimeric Ultralong CDR-H3 Common Light Chain Antibodies Targeting NKp30 and EGFR

All 13 remaining NKp30-specific ultralong CDR-H3 paratopes were subsequently combined with two EGFR-targeting ultralong CDR-H3 antibodies (60F06 and 60H05) we have previously generated (**Supplementary Figure 3**). Notably, both EGFR specific entities represent unique clonotypes, based on sequence similarity of CDR-H3, allowing for a more thorough characterization in terms of biophysical, biochemical as well as functional properties of generated ultralong common light chain bispecifics. For bsAb production, all 13 NKp30 targeting paratopes were expressed on the SEED AG chain, while EGFR-specific clones were genetically fused to the GA chain of the SEED. In addition to both Fc-effector silenced heavy chains, the same chimeric light chain based on V30 exploited for antibody discovery was utilized for common light chain bsAb expression (**Figure 1** and **Supplementary Figure 1**). Following production and single step purification by protein A, all 26 bispecifics were scrutinized in terms of expression yields as well as target monomer species by analytical size exclusion chromatography (SEC), as shown in **Table 1**. Except for two molecules, expression yields post protein A purification were in the double digit milligram per liter scale, which can be considered as acceptable for transient protein production, especially given the high complexity of this kind of bsAb. Of note, tranfections were performed in a 2:1:1 ratio (i.e. AG plasmid: GA plasmid: light chain plasmid). By modifying plasmid ratios for transfection, expression yields might be further optimized. Interestingly, there was a clear trend for higher yields for all bispecific molecules based on EGFR-targeting paratope 60H05 in direct comparison to 60F06. This is highlighting the impact of individual paratopes on protein production. Aggregation properties were determined by analytical SEC and unveiled more than 90% target peak for 24 out of 26 common light chain bispecifics (**Table 1** and **Supplementary Figures 4, 5**). Only 63D06x60H05 with 88.8% target peak and 63H02x60H05 with 88.9% main peak were slightly below this threshold, indicating rather favorable biophysical properties of the herein engineered cattle-derived bsAbs.

**Table 1:** Biophysical and biochemical characterization of cattle-derived ultralong CDR-H3 common light chain bispecific antibodies.

Bispecific molecule	Yield [mg/L]	SEC [%]	KD EGFR [M]	kon EGFR [1/Ms]	koff EGFR [1/s]	KD NKp30 [M]	kon NKp30 [1/Ms]	koff NKp30 [1/s]
63A10x60F06	14,6	97,9	7,4E-09	9,0E+04	6,6E-04	2,0E-08	6,0E+05	1,2E-02
63B08x60F06	11,3	96,3	2,0E-08	6,9E+04	1,4E-03	3,4E-09	1,3E+06	4,3E-03
63B12x60F06	14,5	98,6	1,5E-08	7,1E+04	1,1E-03	2,0E-09	1,4E+06	2,7E-03
63C01x60F06	14,9	96,9	1,4E-08	7,3E+04	1,0E-03	8,5E-08	8,3E+05	7,0E-02
63D02x60F06	14,7	98,5	1,4E-08	6,0E+04	8,4E-04	4,7E-09	1,5E+06	7,1E-03
63D05x60F06	19,2	96,8	6,5E-09	7,9E+04	5,1E-04	9,5E-10	1,9E+06	1,9E-03
63D06x60F06	7,9	94,4	1,0E-08	8,3E+04	8,3E-03	1,1E-08	3,6E+05	4,1E-03
63D10x60F06	9,2	97,8	1,7E-08	7,0E+04	1,2E-03	8,9E-08	4,8E+05	4,2E-02
63E11x60F06	46,1	97,3	1,3E-08	7,9E+04	1,0E-03	1,4E-09	1,9E+05	2,7E-04
63E12x60F06	26,4	100	1,0E-08	7,0E+04	7,3E-04	9,6E-08	6,7E+05	6,5E-02
63F07x60F06	40,0	99,6	9,0E-09	7,1E+04	6,3E-04	8,2E-10	1,3E+06	1,1E-03
63F12x60F06	29,0	99,2	1,0E-08	7,8E+04	7,9E-04	2,3E-08	8,0E+05	1,9E-02
63H02x60F06	10,1	94,5	1,1E-08	8,4E+04	9,5E-04	1,2E-09	1,2E+06	1,4E-03
63A10x60H05	27,0	93,8	2,0E-08	9,1E+04	1,9E-03	1,4E-08	3,0E+05	4,1E-03
63B08x60H05	16,2	93,7	2,3E-08	1,4E+05	3,2E-03	5,4E-09	1,1E+06	6,0E-03
63B12x60H05	16,4	94,4	2,2E-08	7,8E+04	1,7E-03	2,4E-09	1,2E+06	2,8E-03
63C01x60H05	34,4	93,8	1,9E-08	1,0E+05	2,0E-03	6,3E-08	8,2E+05	5,2E-02
63D02x60H05	15,3	95,8	1,9E-08	9,0E+04	1,7E-03	1,6E-09	1,6E+06	2,5E-03
63D05x60H05	30,1	93,8	2,1E-08	9,2E+04	1,9E-03	7,8E-10	1,5E+06	1,2E-03
63D06x60H05	15,1	88,8	1,8E-08	9,6E+04	1,7E-03	1,7E-08	2,7E+05	4,6E-03
63D10x60H05	37,7	92,0	1,9E-08	9,2E+04	1,8E-03	4,4E-08	5,5E+05	2,5E-02
63E11x60H05	63,9	91,9	2,0E-08	1,1E+05	2,1E-03	3,1E-09	2,3E+05	6,9E-04
63E12x60H05	47,2	96,2	1,5E-08	9,7E+04	1,4E-03	6,9E-08	9,0E+05	6,2E-02
63F07x60H05	61,6	99,5	2,2E-08	1,2E+05	2,6E-03	1,3E-09	1,3E+06	1,6E-03
63F12x60H05	44,4	98,0	1,1E-08	1,0E+05	1,1E-03	6,1E-08	4,9E+05	3,0E-02
63H02x60H05	19,1	88,9	2,8E-08	6,7E+04	1,9E-03	2,5E-09	1,1E+06	2,7E-03

**Figure 3:**
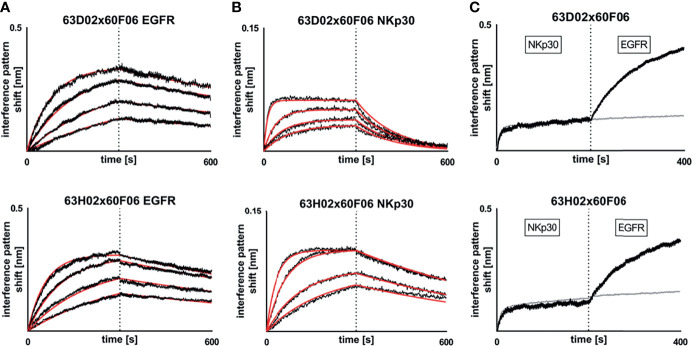
Biochemical characterization of chimeric ultralong CDR-H3 common light chain bispecific antibodies *via* Biolayer interferometry. Kinetic measurements against recombinant human EGFR extracellular protein **(A)** or recombinant human NKp30 ECD **(B)**. Bispecific entities 63D02x60F06 (top) or 63H02x60F06 (bottom) were loaded onto sensor tips. After sensor rinsing, antigen binding was conducted at different concentrations (100 nM, 50 nM, 25 nM and 12.5 nM for EGFR and 50 nM (25 nM for 63H02), 12.5 nM, 6.25 nM and 3.125 nM for NKp30) for 300 s, followed by a dissociation step in kinetics buffer for 300 s. **(C)** Simultaneous binding of 63D02x60F06 (top) or 63H02x60F06 (bottom) bispecifics against NKp30 ECD and EGFR ECD. Bispecifics were loaded to the sensor tips. After sensor rinsing two consecutive association steps were performed at 100 nM (Nkp30) and 100 nM (EGFR) for 200 s each.

Subsequently, we determined binding kinetics to both antigens, EGFR and NKp30, as exemplarily shown for bsAb 63D02x60F06 and 63H02x60F06 in **Figures 3A, B** (**Table 1**, **Figure 3** and **Supplementary Figures 6, 7**). In accordance with affinity measurements conducted previously (31), all bsAbs incorporating EGFR-directed paratope 60H05 specifically bound to recombinant human EGFR ECD in the lower double digit nanomolar range, whereas bsAbs harboring EGFR-specific ultralong CDR-H3 common light chain paratope 60F06 displayed affinities for EGFR in the single digit to lower double digit nanomolar range. Affinities for NKp30 ranged from picomolar to double digit nanomolar binding demonstrating a wide range of affinities of isolated NKp30-binding ultralong CDR-H3 common light chain paratopes. Only minor to moderate differences in kinetics were observed for identical NKp30 binding sites when reformatted with the two different EGFR-addressing paratopes. This is giving some evidence that main binding characteristics remain largely unaffected when individual common light chain ultralong CDR-H3 paratopes are incorporated into different bsAbs. Moreover, all cattle-derived chimeric bsAbs were capable of simultaneously binding to both, EGFR as well as NKp30 recombinant human ECDs (**Figure 3C** and **Supplementary Figures 6, 7**).

### Bispecific Bovine x Human Chimeric Ultralong CDR-H3 Common Light Chain Antibodies Targeting NKp30 and EGFR Elicit Significant NK-Cell Mediated Lysis of EGFR-Overexpressing Tumor Cells as Well as Robust Proinflammatory Cytokine Release

**Figure 4:**
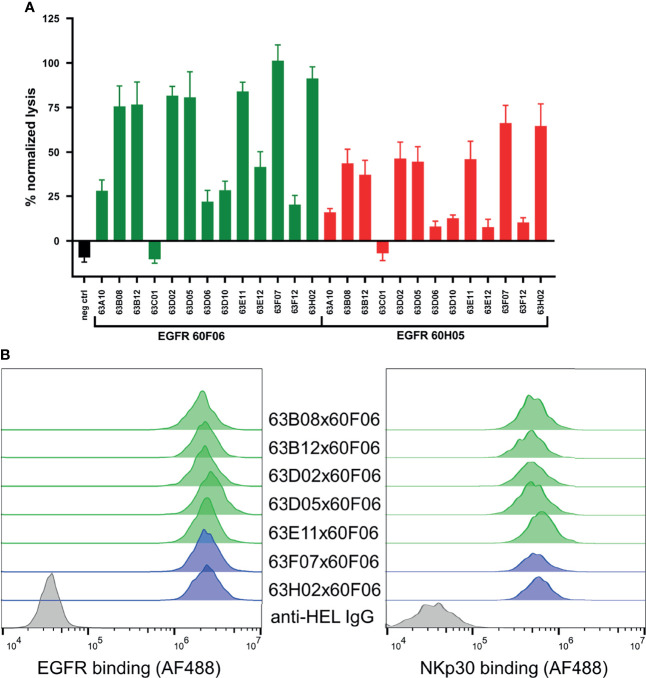
Killing capacities of 26 generated cattle derived common light chain bispecifics **(A)** and cellular binding (**B**, left) as well as simultaneous binding to A431 and NKp30 ECD (**B**, right). Fluorescence-microscopy based killing assay using EGFR-positive A431 target cells and PBMC-purified NK effector cells at an E:T ratio of 5:1 **(A)** as well as cattle derived bsAbs at a concentration of 50 nM. A monospecific EGFR targeting Fc effector silenced negative control was included (black). Individual bsAbs based on EGFR targeting paratope 60F06 shown in green and entities based on EGFR-specific binder 60H05 given in red. Data was normalized to allow comparison of the independent experiments. Graphs show normalized means ± SEM of n = 3 different healthy donors. (**B**, left) Cellular binding of selected 60F06 based cattle-derived ultralong CDR-H3 common light chain bsAbs to EGFR expressing A431 cells at 100 nM. B7-H6 competing molecules shown in green, molecules targeting another epitope on NKp30 given in blue. An anti-HEL IgG control was included (grey). Cellular binding properties were detected *via* a fluorophore conjugated anti-human Fc antibody (**B**, right) Simultaneous binding properties of generated bispecifics. A431 cells were incubated with engineered common light chain bispecifics at 100 nM (green: B7-H6 competitors, blue: B7-H6 non competitors) followed by incubation with his-tagged NKp30 ECD at 200 nM. Simultaneous binding was detected *via* a fluorescence-labeled anti-his antibody.

We also set out to scrutinize whether generated cattle-derived chimeric ultralong common light chain bsAbs could trigger efficient NK cell redirection, resulting in killing of EGFR-overexpressing tumor cells. To this end, we ranked all 26 engineered common light chain bispecifics in a killing assay exploiting EGFR-positive cell line A431 as well as peripheral blood mononuclear cell (PBMC)-isolated NK cells of three healthy donors (**Figure 4A**). All molecules were assessed for their killing capacities at a concentration of 50 nM. This revealed that besides one NKp30-targeting paratope that was unfunctional in combination with both EGFR-directed antigen binding sites (similar to an EGFR-targeting Fc-silenced negative control), 12 out of 13 NKp30-directed binders triggered tumor cell lysis in conjunction with both EGFR-paratopes to some extent (**Figure 4A**). Amongst those, seven NKp30-directed paratopes (63B08, 63B12, 63D02, 63D05, 63E11, 63F07 and 63H02) were robust in eliciting killing of A431 cells. B7-H6, the cell bound ligand of NKp30, is upregulated on tumor cells and absent on most normal cells (42). In this respect, B7-H6 acts as ‘danger signal’ providing a positive input for NK cell activation *via* the NKp30 axis. To characterize epitope coverage more thoroughly, we investigated whether those seven NKp30 targeting cattle derived binders address a similar region as the natural ligand on NKp30 (**Supplementary Figure 8**). Within this set of bispecifics, five NKp30 paratopes competed with B7-H6 for binding to NKp30, while two NKp30 directed antigen binding sites did not show competition (63F07 and 63H02). Interestingly, when reformatted as bispecific together with EGFR-specific binder 60F06 killing capacities were more pronounced than for 60H05 SEEDbodies, clearly indicating dependencies of cytotoxic synapse formation on the tumor targeting antigen binding site of the bispecific molecule.

To get a more profound understanding on NK cell redirection, we focused on the seven NKp30-targeting cattle-derived paratopes that mediated robust killing in initial assays reformatted as bispecific common light chain SEED with 60F06. As expected, all seven cattle-derived common light chain bsAbs showed specific binding to EGFR-overexpressing tumor cell line A431 with similar mean fluorescence intensities (**Figure 4B**, left). Additionally, we set out to assess simultaneous binding on the cellular level. On PBMC-derived NK cells, NKp30 is only expressed at very low levels with approximately 1000 molecules per cell (34). Due to this, binding to NKp30 is hardly detectable *via* flow cytometry (33). To this end, we exploited an indirect binding assay to detect simultaneous binding. At first, EGFR-positive A431 cells were coated with cattle-derived ultralong CDR-H3 common light chain bsAbs. Subsequently, his-tagged NKp30 ECD was added and simultaneous binding was monitored *via* application of a his-tag specific fluorophore-coupled detection antibody. This resulted in specific interactions of NKp30 with A431 cells for all seven bispecific molecules (**Figure 4B**, right). Hence, all bispecifics bound simultaneously to cell surface expressed EGFR and the soluble form of trigger receptor NKp30.

**Figure 5:**
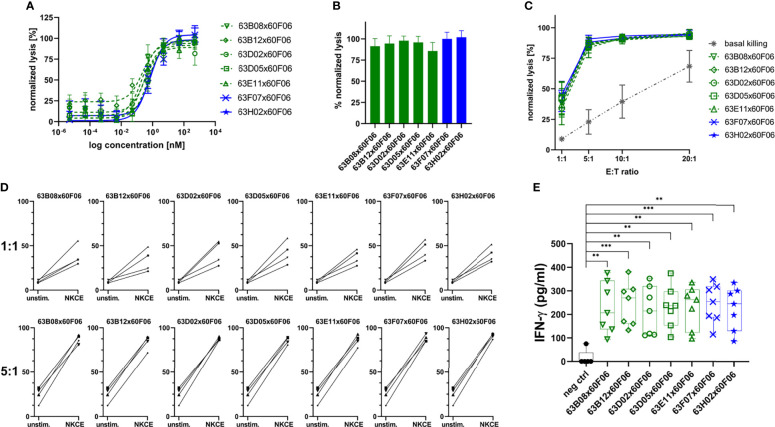
Characterization of selected cattle-derived ultralong CDR-H3 common light chain bsAbs in terms of killing capacities and cytokine release. Fluorescence-microscopy based killing assay using EGFR-positive A431 target cells and PBMC-purified NK effector cells at an E:T ratio of 5:1. Analysis of dose-dependent **(A)** and maximum **(B)** target cell killing. B7H6 competitors shown in green, B7-H6 non competitors given in blue. Data was normalized to allow comparison of the independent experiments. Graphs show normalized means ± SEM of n = 8 different healthy donors. **(C)** Maximum killing capacities at different effector to target (E:T) ratios. Cattle derived common light chain bsAbs were applied at a concentration of 50 nM. Grey: basal killing activities of NK cells i.e. without addition of bsabs. Data was normalized to allow comparison of the independent experiments. Graphs show normalized means ± SEM of n = 3-4 different healthy donors. **(D)** Donor specific lysis capacities at an E:T ratio of 1:1 and 5:1. BsAbs were added at 50 nM (NKCE). Unstim: Basal killing of NK cells in the absence of bsAbs. Data was normalized to allow comparison of the independent experiments. Graphs show normalized means ± SEM of n = 4 different healthy donors. **(E)** NK cell-mediated IFN-γ using cytokine HTRF kits for quantification. Purified NK cells were co-cultured with A431 cells for 24 h at an E:T ratio of 5:1 prior to analysis. Graphs show box and whisker plots as superimposition with dot plots of 7 individual experiments. *** p≤0.001, ** p≤0.01. B7-H6 competing molecules shown in green, molecules targeting another epitope on NKp30 given in blue.

Afterwards, those molecules were assessed more meticulously in killing assays using PBMC-isolated NK cells of eight healthy donors in a dose-response curve ranging from 0.005 pM to 500 nM (**Figure 5A** and **Table 2**). All seven common light chain bsAbs triggered significant lysis of EGFR-overexpressing A431 cells in a dose-dependent manner with potencies (EC_50_killing) in the picomolar range. In this regard, potencies ranged from 219 pM for 63B08x60F06 to 807 pM for 63H02x60F06 and also efficacies were similar among this selected set of cattle-derived ultralong CDR-H3 bispecifics. In this regard, potencies ranged from 219 pM for 63B08x60F06 to 807 pM for 63H02x60F06 and also efficacies were similar among this selected set of cattle-derived ultralong CDR-H3 bispecifics (**Figure 5B**). Additionally, for none of those molecules we observed significant NK cell mediated killing of EGFR-negative CHO cells (**Supplementary Figure 9**).

**Table 2:** NK cell mediated target cell dependent cytotoxic properties and IFN-γ release triggered by selected cattle derived ultralong CDR-H3 common light chain bispecific antibodies.

Bispecific molecule	EC50 killing [pM]	Max killing [%]	Mean IFN-γ release [pg/mL]
63B08x60F06	219	91.4	229.3
63B12x60F06	225	94.5	245.1
63D02x60F06	325	98.0	214.4
63D05x60F06	540	95.9	231.0
63E11x60F06	273	85.8	232.0
63F07x60F06	598	100	245.6
63H02x60F06	807	102	226.1

Furthermore, cytotoxic capacities of engineered ultralong CDR-H3 common light chain bsAbs were scrutinized at varying effector cell (i.e. NK cell) to target cell ratios (**Figure 5C**). In line with their natural ability to spontaneously lyse tumor cells (43, 44), basal killing activities, i.e. killing without bsAb redirection of NK cells were significantly amplified by increasing E:T ratios. For all assessed constructs, NK cells efficiently triggered lysis of EGFR-overexpressing cells even at low ratios. Efficacies in the presence of bsAbs were substantially higher compared to NK cell mediated tumor cell lysis alone throughout all different settings. In this respect, nearly half-maximal lysis was already observed at a 1:1 E:T ratio under saturating conditions. At a 5:1 E:T ratio maximal overall lysis was almost achieved and higher ratios only minorly affected efficacies. Most importantly, the different generated bispecifics did not behave appreciably different under these variable conditions. Next, we analyzed redirection capabilities of the different bsAbs on the individual donor level to get a glimpse on donor to donor variation (**Figure 5D**). At an E:T ratio of 5:1, all seven distinct cattle-derived ultralong CDR-H3 bispecifics behaved quite similar with only subtle differences in eliciting lysis of EGFR-overexpressing tumor cells by PBMC-isolated NK cells from individual healthy donors. Moreover, variations in donor to donor responses were barely neglectable. Intriguingly, when lowering the E:T ratio to 1:1, differences were quite more profound. While again the different bsAbs behaved overall rather similar, NK cells from individual donors triggered considerably different maximum killing levels under saturating conditions of applied antibodies. In this respect, efficacies varied between approximately 20% lysis to more than 50% killing. Essentially, all seven generated bispecifics were able to efficiently trigger lysis of A431 cells by redirecting NK cells from all individual donors at low E:T ratios.

Finally, we also looked at the targeted release of IFN-γ as an *in vitro* indicator of a potential targeted inflammation of tumors. To this end, A431 cells were incubated with NK cells either in the presence of cattle-derived common light chain bsAbs or in the presence of an EGFR-targeting Fc-silenced control molecule (**Figure 5E**). All ultralong CDR-H3 bispecifics robustly induced effector-type cytokine production of IFN-γ (**Table 2**). In contrast to this, only negligible levels were detected for the control molecule when added to co-cultured A431 and NK cells. Additionally, only a minor release of IFN-γ was detected when the bispecific molecules were added to EGFR-negative CHO cells that were co-cultured with PBMC-derived NK cells (**Supplementary Figure 10**).

## Discussion

Bispecific antibodies pave the way for completely novel modes of action and consequently emerged as promising molecules for therapeutic intervention (9, 15). To investigate whether cattle-derived ultralong CDR-H3 paratopes can be efficiently engineered into bispecific antibody formats, we have generated bovine ultralong CDR-H3 paratopes directed against NKp30 that share the same light chain with EGFR-specific ultralong CDR-H3 paratopes we have previously isolated and characterized (31). Fascinatingly, in cattle, ultralong CDR-H3 heavy chains typically pair with a single VL gene, VL30 that is relatively sequence conserved (25). As such, bovine ultralong antibodies can be almost considered as a natural source of common light chain paratopes. In general, it has been shown that this non-classical type of immunoglobulin is predisposed to address epitopes that might only be inefficiently targeted by conventional antibodies. Stanfield and colleagues, for instance, demonstrated that a broadly neutralizing anti-HIV ultralong CDR-H3 paratope hits an epitope on the gp120 CD4 binding site (25) that is typically recessed for conventional antibodies (45). Hence, it is tempting to speculate that ultralong CDR-H3 antibodies might enlarge the ‘druggable’ target space.

For the isolation of ultralong CDR-H3 entities we have specifically amplified this region from the PBMC-repertoire of immunized cattle and engrafted it onto a fixed chimeric Fab heavy chain that was paired with a single VL30 region we have previously used for the isolation of bovine ultralong EGFR-specific antibodies (31). By exploiting yeast surface display, NKp30-specific antibodies were readily obtained within two rounds of FACS sorting and eventually, after soluble antibody expression, 13 unique clones showed specific binding to this target. Subsequently, those were reformatted as bispecific common light chain antibodies with two different ultralong CDR-H3 paratopes directed against EGFR by employing the same light chain that has been used for YSD. Essentially, the vast majority of the generated 26 molecules showed ‘early signs’ of favorable biophysical properties as well as simultaneous binding to both antigens on the protein level, a prerequisite for effector cell recruitment. When the same NKp30 targeting cattle derived ultralong CDR-H3 common light chain paratopes were reformatted into bsAbs harboring different EGFR directed paratopes, we observed only minor to moderate differences in affinities. Albeit an impact of the EGFR based ultralong CDR-H3 paratope cannot be entirely excluded, this is indicating that main binding properties of these antigen binding sites remain mostly unaffected when produced as asymmetric common light chain bsAb. Positioning effects resulting in impaired affinities have been described for multiple different bispecific antibody platforms (18, 46–49). It will be interesting to investigate whether such effects can be observed for cattle-derived ultralong CDR-H3 common light chain binders, when reformatted into more complex formats.

In a first killing assay, 24 out of 26 bispecific common light chain derivatives mediated significant NK cell mediated lysis of EGFR-overexpressing A431 cells with one NKp30-targeting paratope remaining unfunctional when combined with both EGFR-directed paratopes. Maximum killing was more pronounced for NKp30-directed clones when reformatted with EGFR-binder 60F06. Interestingly, 60F06 seems to target the same subdomain on the ECD of EGFR as 60H05 but does not compete with it for binding to EGFR (31). Hence, it targets a different epitope. In general, differences in killing capacities of the generated molecules are not unexpected, given the multiple parameters that have a major impact on cytolytic synapse formation of effector cells (including synapse distance as well as the epitopes that are targeted on the tumor associated antigen as well as on the effector trigger molecule) (50). Most importantly, all seven bsAbs that were scrutinized more meticulously in terms of killing abilities elicited robust NK cell mediated lysis of tumor cells in a targeted fashion with negligible killing of EGFR-negative cells. Potencies were in the picomolar range for all the molecules tested. As such, cytotoxic capacities were similar to those reported for NK cell engagers based on the natural ligand of NKp30 referred to as B7-H6 or affinity optimized versions thereof (32–34). Only at a low E:T ratio of 1:1 we observed differential killing on the single donor level i.e. donor to donor variations. Notwithstanding, significant killing was observed for all the donors tested in this particular setting. Furthermore, at higher E:T ratios all donors behaved quite similar in robustly triggering NK cell mediated killing of tumor cells. Variations in maximum lysis on the donor to donor level have been previously reported for NK cell engagers by Peipp and co-workers (34). For their HER2-specific bifunctional immunoligand harboring B7-H6, differences in efficacies were even observed at higher E:T ratios of 10:1. Finally, all cattle-derived common light chain bispecific compounds significantly triggered the release of IFN-γ in a strictly tumor cell targeted manner. Considering the multiple pleiotropic effects of IFN-γ such as inhibiting suppressive immune cell subsets (51, 52) and NK, NKT and T cell trafficking into tumors through the induction of chemokine production (53), this might be envisioned to result into a targeted inflammation of tumors when applied *in vivo*. In conclusion, our data suggest that cattle-derived ultralong CDR-H3 paratopes enable the facile generation of common light chain bispecifics suitable for effector cell redirection.

## Data Availability Statement

The original contributions presented in the study are included in the article/**Supplementary Material**. Further inquiries can be directed to the corresponding author.

## Ethics Statement

The animal study was reviewed and approved by Niedersächsisches Landesamt für Verbraucherschutz und Lebensmittelsicherheit (LAVES), Dezernat 33 – Tierschutzdienst. Number: 33.19-42502-05-17A210.

## Author Contributions

SZ, HK, and LP conceived and designed the experiments. LP, PA, and DK performed experiments. LP, DK, PA, and SZ analyzed the data. SZ and LP wrote the manuscript. SK and BV gave scientific advice. All authors contributed to the article and approved the manuscript.

## Conflict of Interest

Authors SZ, LP, SK, PA, and BV are employees of Merck Healthcare KGaA. Author DK was taking part in an internship at Merck Healthcare KGaA at the time of this study.

The remaining author declares that the research was conducted in the absence of any commercial or financial relationships that could be construed as a potential conflict of interest

## Publisher’s Note

All claims expressed in this article are solely those of the authors and do not necessarily represent those of their affiliated organizations, or those of the publisher, the editors and the reviewers. Any product that may be evaluated in this article, or claim that may be made by its manufacturer, is not guaranteed or endorsed by the publisher.

## References

[1] de Taeye SW Rispens T Vidarsson G. The Ligands for Human IgG and Their Effector Functions. Antibodies (2019) 8:30. doi: 10.3390/antib8020030 PMC6640714 31544836

[2] Chiu ML Goulet DR Teplyakov A Gilliland GL. Antibody Structure and Function: The Basis for Engineering Therapeutics. Antibodies (2019) 8:55. doi: 10.3390/antib8040055 PMC6963682 31816964

[3] Mullard A. FDA Approves 100th Monoclonal Antibody Product. Nat Rev Drug Discovery (2021) 20(7):491–495. doi: 10.1038/d41573-021-00079-7 33953368

[4] Wu C Ying H Grinnell C Bryant S Miller R Clabbers A. Simultaneous Targeting of Multiple Disease Mediators by a Dual-Variable-Domain Immunoglobulin. Nat Biotechnol (2007) 25:1290–7. doi: 10.1038/nbt1345 17934452

[5] Krah S Sellmann C Rhiel L Schröter C Dickgiesser S Beck J. Engineering Bispecific Antibodies With Defined Chain Pairing. New Biotechnol (2017) 39:167–73. doi: 10.1016/j.nbt.2016.12.010 28137467

[6] Brinkmann U Kontermann RE. Bispecific Antibodies. Science (2021) 372:916–7. doi: 10.1126/science.abg1209 34045345

[7] Sheridan C. Bispecific Antibodies Poised to Deliver Wave of Cancer Therapies. Nat Biotechnol (2021) 39:251–4. doi: 10.1038/s41587-021-00850-6 33692520

[8] Amivantamab OK'd for EGFR-Mutant NSCLC. Cancer Discov (2021) 11(7):1604. doi: 10.1158/2159-8290.CD-NB2021-0351 34083225

[9] Labrijn AF Janmaat ML Reichert JM Parren PWHI. Bispecific Antibodies: A Mechanistic Review of the Pipeline. Nat Rev Drug Discov (2019) 18:585–608. doi: 10.1038/s41573-019-0028-1 31175342

[10] Nie S Wang Z Moscoso-Castro M D’Souza P Lei C Xu J. Biology Drives the Discovery of Bispecific Antibodies as Innovative Therapeutics. Antibody Ther (2020) 3:18–62. doi: 10.1093/abt/tbaa003 PMC7990219 33928225

[11] Krah S Kolmar H Becker S Zielonka S. Engineering IgG-Like Bispecific Antibodies—An Overview. Antibodies (2018) 7:28. doi: 10.3390/antib7030028 PMC6640676 31544880

[12] Ward E. VH Shuffling can be Used to Convert an Fv Fragment of Anti-Hen Egg Lysozyme Specificity to One That Recognizes a T Cell Receptor Vα. Mol Immunol (1995) 32:147–56. doi: 10.1016/0161-5890(94)00119-L 7870066

[13] Bogen JP Hinz SC Grzeschik J Ebenig A Krah S Zielonka S. Dual Function pH Responsive Bispecific Antibodies for Tumor Targeting and Antigen Depletion in Plasma. Front Immunol (2019) 10:1892. doi: 10.3389/fimmu.2019.01892 31447859 PMC6697062

[14] Rosowski S Becker S Toleikis L Valldorf B Grzeschik J Demir D. A Novel One-Step Approach for the Construction of Yeast Surface Display Fab Antibody Libraries. Microb Cell Factories (2018) 17(1):3. doi: 10.1186/s12934-017-0853-z PMC5759264 29316915

[15] Brinkmann U Kontermann RE. The Making of Bispecific Antibodies. mAbs (2017) 9:182–212. doi: 10.1080/19420862.2016.1268307 28071970 PMC5297537

[16] Chanier T Chames P. Nanobody Engineering: Toward Next Generation Immunotherapies and Immunoimaging of Cancer. Antibodies (2019) 8:13. doi: 10.3390/antib8010013 PMC6640690 31544819

[17] Jovčevska I Muyldermans S. The Therapeutic Potential of Nanobodies. BioDrugs (2019) 34(1):11–26. doi: 10.1007/s40259-019-00392-z PMC6985073 31686399

[18] Pekar L Busch M Valldorf B Hinz SC Toleikis L Krah S. Biophysical and Biochemical Characterization of a VHH-Based IgG-Like Bi- and Trispecific Antibody Platform. mAbs (2020) 12(1):1812210. doi: 10.1080/19420862.2020.1812210 32887531 PMC7531565

[19] Ubah OC Buschhaus MJ Ferguson L Kovaleva M Steven J Porter AJ. Next-Generation Flexible Formats of VNAR Domains Expand the Drug Platform’s Utility and Developability. Biochem Soc Trans (2018) 46:1559–65. doi: 10.1042/BST20180177 30381336

[20] Zielonka S Empting M Grzeschik J Könning D Barelle CJ Kolmar H. Structural Insights and Biomedical Potential of IgNAR Scaffolds From Sharks. mAbs (2015) 7:15–25. doi: 10.4161/19420862.2015.989032 25523873 PMC4622739

[21] Könning D Zielonka S Grzeschik J Empting M Valldorf B Krah S. Camelid and Shark Single Domain Antibodies: Structural Features and Therapeutic Potential. Curr Opin Struct Biol (2017) 45:10–6. doi: 10.1016/j.sbi.2016.10.019 27865111

[22] Haakenson JK Huang R Smider VV. Diversity in the Cow Ultralong CDR H3 Antibody Repertoire. Front Immunol (2018) 9:1262. doi: 10.3389/fimmu.2018.01262 29915599 PMC5994613

[23] Deiss TC Vadnais M Wang F Chen PL Torkamani A Mwangi W. Immunogenetic Factors Driving Formation of Ultralong VH CDR3 in Bos Taurus Antibodies. Cell Mol Immunol (2019) 16:53–64. doi: 10.1038/cmi.2017.117 29200193 PMC6318308

[24] Dong J Finn JA Larsen PA Smith TPL Crowe JE. Structural Diversity of Ultralong CDRH3s in Seven Bovine Antibody Heavy Chains. Front Immunol (2019) 10:558. doi: 10.3389/fimmu.2019.00558 30967877 PMC6440498

[25] Stanfield RL Berndsen ZT Huang R Sok D Warner G Torres JL. Structural Basis of Broad HIV Neutralization by a Vaccine-Induced Cow Antibody. Sci Adv (2020) 6:eaba0468. doi: 10.1126/sciadv.aba0468 32518821 PMC7253169

[26] Wang F Ekiert DC Ahmad I Yu W Zhang Y Bazirgan O. Reshaping Antibody Diversity. Cell (2013) 153:1379–93. doi: 10.1016/j.cell.2013.04.049 PMC4007204 23746848

[27] Stanfield RL Wilson IA Smider VV. Conservation and Diversity in the Ultralong Third Heavy-Chain Complementarity-Determining Region of Bovine Antibodies. Sci Immunol (2016) 1:aaf7962–aaf7962. doi: 10.1126/sciimmunol.aaf7962 27574710 PMC5000368

[28] Cai W-Q Zeng L-S Wang L-F Wang Y-Y Cheng J-T Zhang Y. The Latest Battles Between EGFR Monoclonal Antibodies and Resistant Tumor Cells. Front Oncol (2020) 10:1249. doi: 10.3389/fonc.2020.01249 32793499 PMC7393266

[29] Seshacharyulu P Ponnusamy MP Haridas D Jain M Ganti AK Batra SK. Targeting the EGFR Signaling Pathway in Cancer Therapy. Expert Opin Ther Targets (2012) 16:15–31. doi: 10.1517/14728222.2011.648617 22239438 PMC3291787

[30] Guardiola S Varese M Sánchez-Navarro M Giralt E. A Third Shot at EGFR: New Opportunities in Cancer Therapy. Trends Pharmacol Sci (2019) 40:941–55. doi: 10.1016/j.tips.2019.10.004 31706618

[31] Pekar L Klewinghaus D Arras P Carrara SC Harwardt J Krah S. Milking the Cow: Cattle-Derived Chimeric Ultralong CDR-H3 Antibodies and Their Engineered CDR-H3-Only Knobbody Counterparts Targeting Epidermal Growth Factor Receptor Elicit Potent NK Cell-Mediated Cytotoxicity. Front Immunol (2021) 12:4378. doi: 10.3389/fimmu.2021.742418 PMC8573386 34759924

[32] Pekar L Klausz K Busch M Valldorf B Kolmar H Wesch D. Affinity Maturation of B7-H6 Translates Into Enhanced NK Cell–Mediated Tumor Cell Lysis and Improved Proinflammatory Cytokine Release of Bispecific Immunoligands *via* NKp30 Engagement. J Immunol (2021) 206:225–36. doi: 10.4049/jimmunol.2001004 PMC7750860 33268483

[33] Kellner C Maurer T Hallack D Repp R van de Winkel JGJ Parren PWHI. Mimicking an Induced Self Phenotype by Coating Lymphomas With the NKp30 Ligand B7-H6 Promotes NK Cell Cytotoxicity. J Immunol (2012) 189:5037–46. doi: 10.4049/jimmunol.1201321 23066150

[34] Peipp M Derer S Lohse S Staudinger M Klausz K Valerius T. HER2-Specific Immunoligands Engaging NKp30 or NKp80 Trigger NK-Cell-Mediated Lysis of Tumor Cells and Enhance Antibody-Dependent Cell-Mediated Cytotoxicity. Oncotarget (2015) 6(31):32075–88. doi: 10.18632/oncotarget.5135 PMC4741660 26392331

[35] Valldorf B Hinz SC Russo G Pekar L Mohr L Klemm J. Antibody Display Technologies: Selecting the Cream of the Crop. Biol Chem (2021). doi: 10.1515/hsz-2020-0377 33759431

[36] Doerner A Rhiel L Zielonka S Kolmar H. Therapeutic Antibody Engineering by High Efficiency Cell Screening. FEBS Lett (2014) 588:278–87. doi: 10.1016/j.febslet.2013.11.025 24291259

[37] Davis JH Aperlo C Li Y Kurosawa E Lan Y Lo K-M. SEEDbodies: Fusion Proteins Based on Strand-Exchange Engineered Domain (SEED) CH3 Heterodimers in an Fc Analogue Platform for Asymmetric Binders or Immunofusions and Bispecific Antibodies†. Protein Eng Design Select (2010) 23:195–202. doi: 10.1093/protein/gzp094 20299542

[38] Benatuil L Perez JM Belk J Hsieh C-M. An Improved Yeast Transformation Method for the Generation of Very Large Human Antibody Libraries. Protein Eng Design Select (2010) 23:155–9. doi: 10.1093/protein/gzq002 20130105

[39] Roth L Grzeschik J Hinz SC Becker S Toleikis L Busch M. Facile Generation of Antibody Heavy and Light Chain Diversities for Yeast Surface Display by Golden Gate Cloning. Biol Chem (2019) 400:383–93. doi: 10.1515/hsz-2018-0347 30465712

[40] Weaver-Feldhaus JM Lou J Coleman JR Siegel RW Marks JD Feldhaus MJ. Yeast Mating for Combinatorial Fab Library Generation and Surface Display. FEBS Lett (2004) 564:24–34. doi: 10.1016/S0014-5793(04)00309-6 15094038

[41] Schlothauer T Herter S Koller CF Grau-Richards S Steinhart V Spick C. Novel Human IgG1 and IgG4 Fc-Engineered Antibodies With Completely Abolished Immune Effector Functions. Protein Eng Design Select (2016) 29:457–66. doi: 10.1093/protein/gzw040 27578889

[42] Brandt CS Baratin M Yi EC Kennedy J Gao Z Fox B. The B7 Family Member B7-H6 Is a Tumor Cell Ligand for the Activating Natural Killer Cell Receptor NKp30 in Humans. J Exp Med (2009) 206:1495–503. doi: 10.1084/jem.20090681 PMC2715080 19528259

[43] Gonzalez-Rodriguez AP Villa-Álvarez M Sordo-Bahamonde C Lorenzo-Herrero S Gonzalez S. NK Cells in the Treatment of Hematological Malignancies. J Clin Med (2019) 8:1557. doi: 10.3390/jcm8101557 PMC6832953 31569769

[44] Kiessling R Klein E Pross H Wigzell H. “Natural” Killer Cells in the Mouse. II. Cytotoxic Cells With Specificity for Mouse Moloney Leukemia Cells. Characteristics of the Killer Cell. Eur J Immunol (1975) 5:117–21. doi: 10.1002/eji.1830050209 1086218

[45] Sok D Le KM Vadnais M Saye-Francisco KL Jardine JG Torres JL. Rapid Elicitation of Broadly Neutralizing Antibodies to HIV by Immunization in Cows. Nature (2017) 548:108–11. doi: 10.1038/nature23301 PMC5812458 28726771

[46] Wu C Ying H Bose S Miller R Medina L Santora L. Molecular Construction and Optimization of Anti-Human IL-1α/β Dual Variable Domain Immunoglobulin (DVD-Ig ™) Molecules. mAbs (2009) 1:339–47. doi: 10.4161/mabs.1.4.8755 PMC2726605 20068402

[47] DiGiammarino EL Harlan JE Walter KA Ladror US Edalji RP Hutchins CW. Ligand Association Rates to the Inner-Variable-Domain of a Dual-Variable-Domain Immunoglobulin are Significantly Impacted by Linker Design. mAbs (2011) 3:487–94. doi: 10.4161/mabs.3.5.16326 PMC3225853 21814039

[48] Metz S Panke C Haas AK Schanzer J Lau W Croasdale R. Bispecific Antibody Derivatives With Restricted Binding Functionalities That Are Activated by Proteolytic Processing. Protein Eng Design Select (2012) 25:571–80. doi: 10.1093/protein/gzs064 PMC3449404 22976197

[49] Mayer K Baumann A-L Grote M Seeber S Kettenberger H Breuer S. TriFabs—Trivalent IgG-Shaped Bispecific Antibody Derivatives: Design, Generation, Characterization and Application for Targeted Payload Delivery. Int J Mol Sci (2015) 16:27497–507. doi: 10.3390/ijms161126037 PMC4661895 26593903

[50] Chen W Yang F Wang C Narula J Pascua E Ni I. One Size Does Not Fit All: Navigating the Multi-Dimensional Space to Optimize T-Cell Engaging Protein Therapeutics. mAbs (2021) 13:1871171. doi: 10.1080/19420862.2020.1871171 33557687 PMC7889206

[51] Overacre-Delgoffe AE Chikina M Dadey RE Yano H Brunazzi EA Shayan G. Interferon-γ Drives T Reg Fragility to Promote Anti-Tumor Immunity. Cell (2017) 169:1130–1141.e11. doi: 10.1016/j.cell.2017.05.005 28552348 PMC5509332

[52] Medina-Echeverz J Haile LA Zhao F Gamrekelashvili J Ma C Métais J-Y. IFN-γ Regulates Survival and Function of Tumor-Induced CD11b ^+^ Gr-1 ^High^ Myeloid Derived Suppressor Cells by Modulating the Anti-Apoptotic Molecule Bcl2a1: Immunomodulation. Eur J Immunol (2014) 44:2457–67. doi: 10.1002/eji.201444497 PMC4140991 24810636

[53] Groom JR Luster AD. CXCR3 Ligands: Redundant, Collaborative and Antagonistic Functions. Immunol Cell Biol (2011) 89:207–15. doi: 10.1038/icb.2010.158 PMC3863330 21221121

