# Supplementary material for: Grabbing the Bull by Both Horns: Bovine Ultralong CDR-H3 Paratopes Enable Engineering of ‘Almost Natural’ Common Light Chain Bispecific Antibodies Suitable For Effector Cell Redirection
Source: Front Immunol. 2022 Jan 11;12:801368. doi: 10.3389/fimmu.2021.801368 (PMC8787767; doi:10.3389/fimmu.2021.801368)
Supplement: Supplementary file 1 [file DataSheet_1.pdf]

Supplementary Material

1. Supplementary Figures

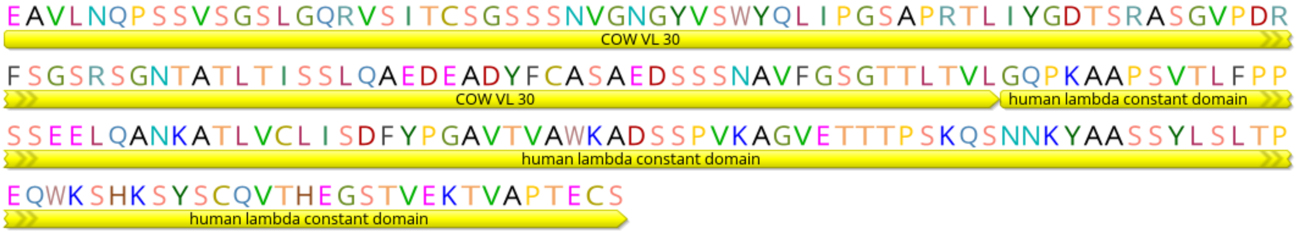

Supplementary Figure 1. Amino acid sequence of bovine variable domain VL30 fused to human constant region CLλ. Figure generated with Geneious Prime® v2021.1.1.

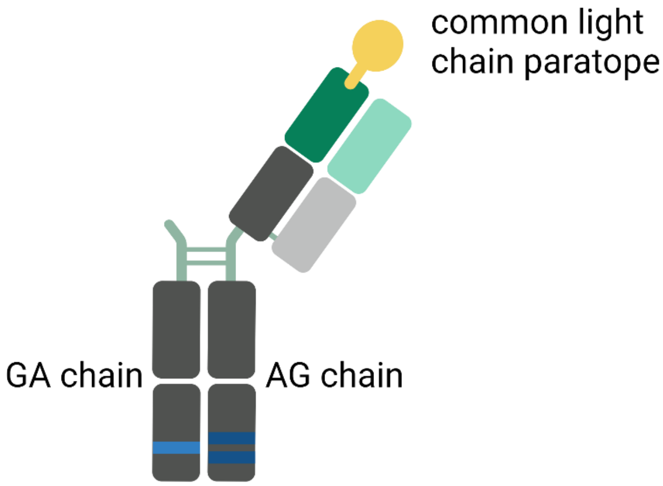

Supplementary Figure 2. Scheme of one armed SEED molecules harboring cattle-derived ultralong CDR-H3 paratopes directed against NKp30. Selected paratopes were grafted onto the AG chain of the SEED molecule (indicated by two dark blue lanes in the CH3 region), while the Fc region of the GA chain was utilized without paratope (indicated by one lane in light blue). Yellow: NKp30-targeting CDR-H3 architectures that were incorporated into a fixed cattle derived VH domain based on IGHV1-7 (dark green). Bovine VL30 (light green) was exploited as common light chain. Human heavy chain constant regions shown in dark grey. Human CLλ given in light grey. Figure generated with biorender (www.biorender.com).

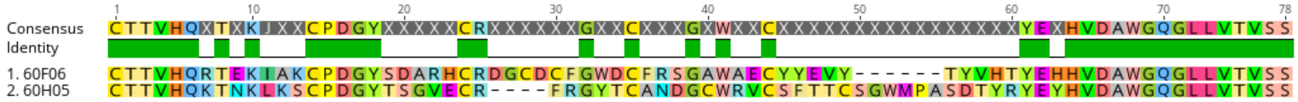

Supplementary Figure 3. CDR-H3 alignment of EGFR-specific ultralong CDR-H3 paratopes utilized in this study. Sequence of IGHJ2-4 is also shown. Amino acids given in 1-letter code and in different colors. Alignment generated with Geneious Prime® v2021.1.1.

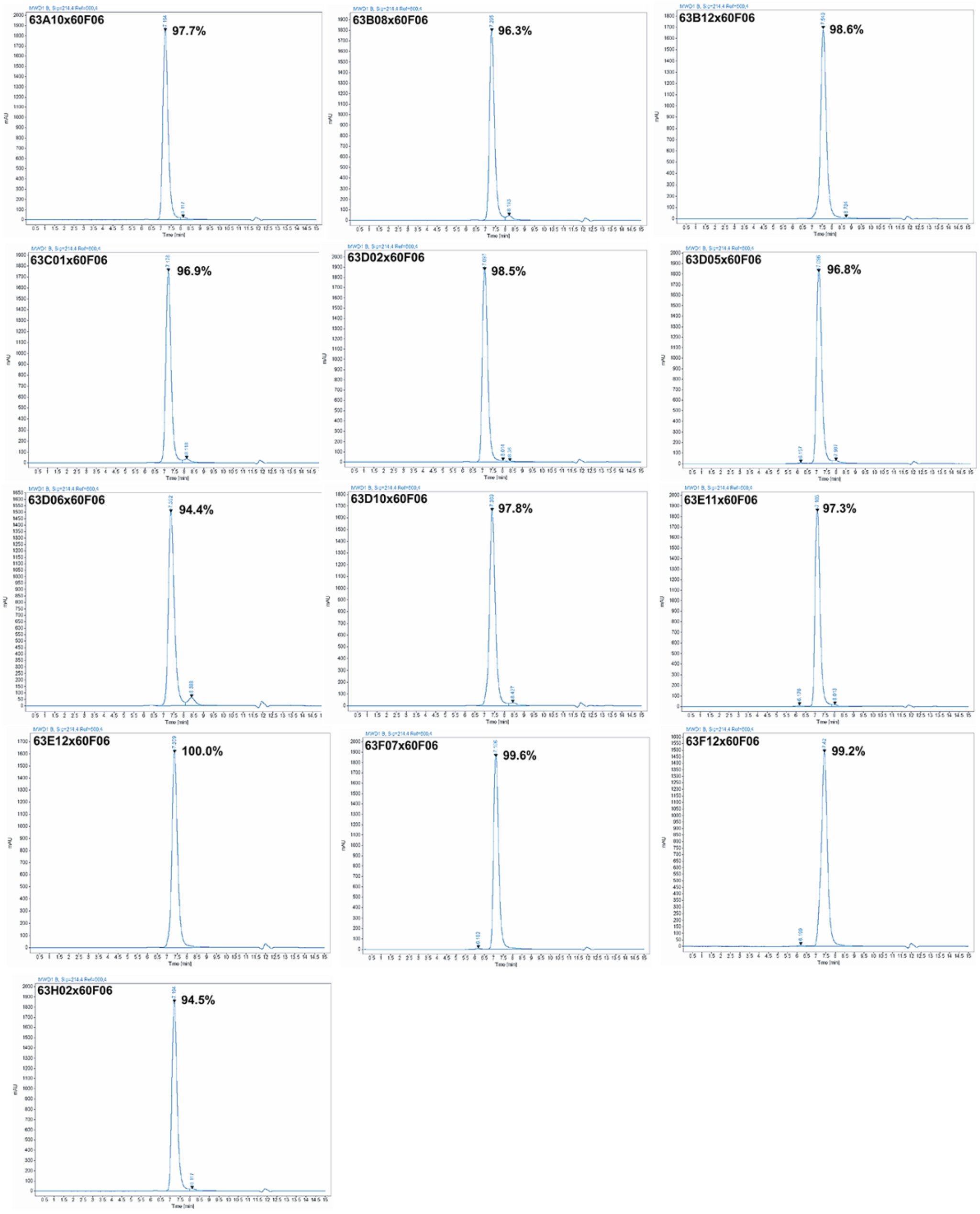

Supplementary Figure 4. Analytical size exclusion chromatography profiles of generated EGFRxNKp30 specific chimeric cattle-derived ultralong CDR-H3 common light chain bsAbs based on EGFR paratope 60F06.

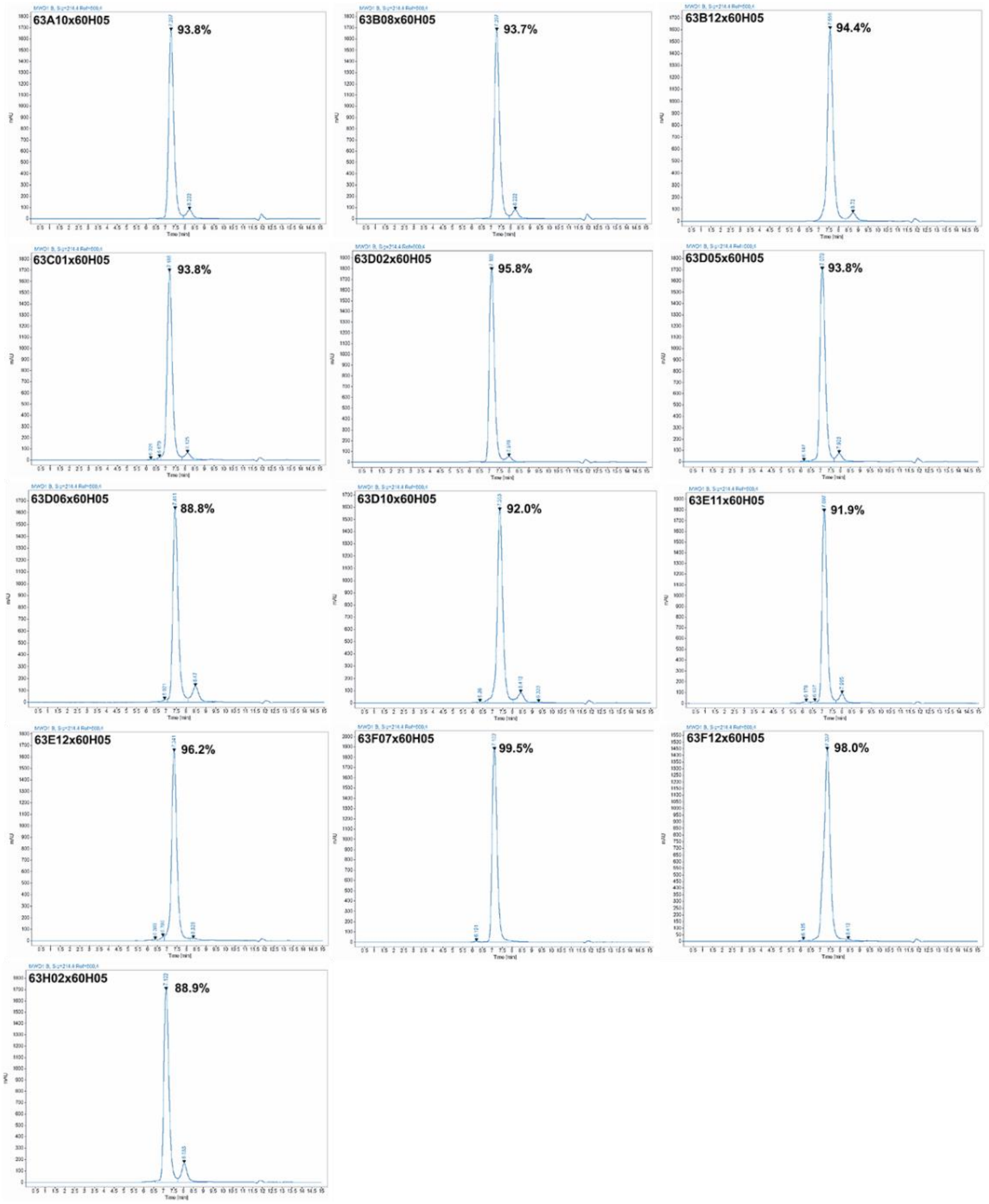

Supplementary Figure 5. Analytical size exclusion chromatography profiles of generated EGFRxNKp30 specific chimeric cattle-derived ultralong CDR-H3 common light chain bispecifics based on EGFR paratope 60H05.

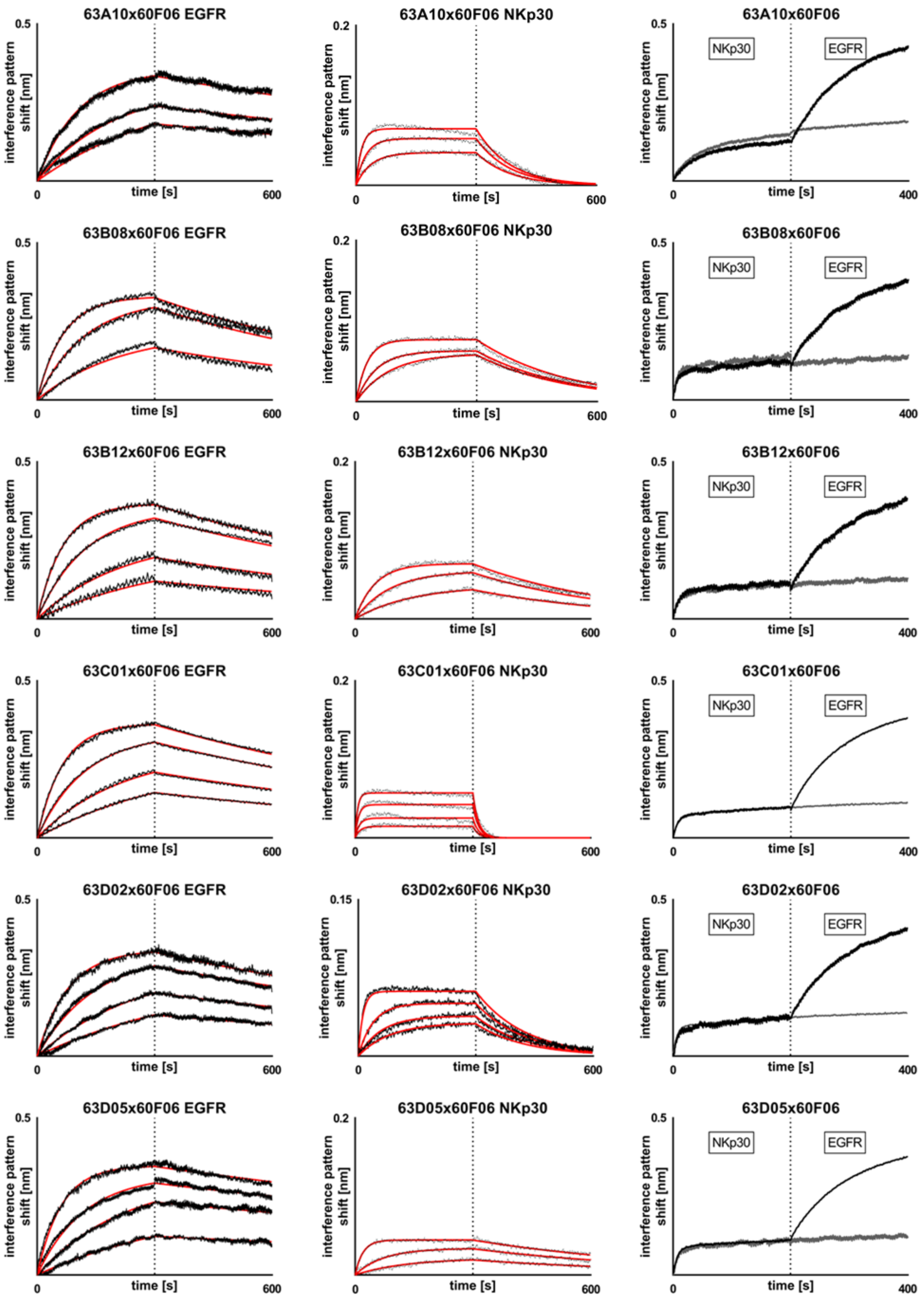
63A10x60F06 EGFR
63A10x60F06 NKp30
63A10x60F06
63B08x60F06 EGFR
63B08x60F06 NKp30
63B08x60F06
63B12x60F06 EGFR
63B12x60F06 NKp30
63B12x60F06
63C01x60F06 EGFR
63C01x60F06 NKp30
63C01x60F06
63D02x60F06 EGFR
63D02x60F06 NKp30
63D02x60F06
63D05x60F06 EGFR
63D05x60F06 NKp30
63D05x60F06
interference pattern shift [nm]
time [s]
NKp30
EGFR
0.5
0.2
0.15
0
600
400

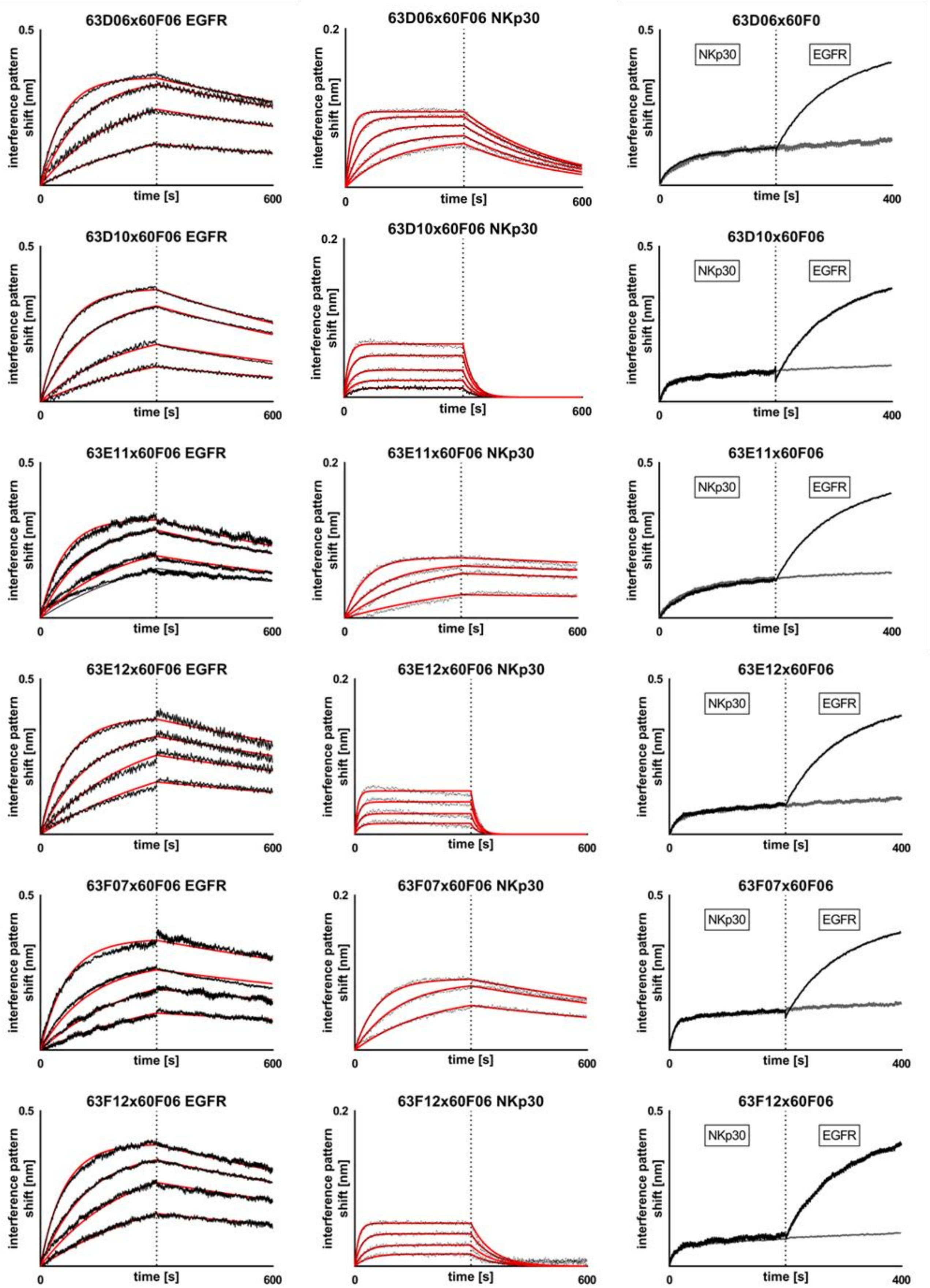
63D06x60F06 EGFR
63D06x60F06 NKp30
63D06x60F0
63D10x60F06 EGFR
63D10x60F06 NKp30
63D10x60F06
63E11x60F06 EGFR
63E11x60F06 NKp30
63E11x60F06
63E12x60F06 EGFR
63E12x60F06 NKp30
63E12x60F06
63F07x60F06 EGFR
63F07x60F06 NKp30
63F07x60F06
63F12x60F06 EGFR
63F12x60F06 NKp30
63F12x60F06
NKp30
EGFR
interference pattern shift [nm]
time [s]
0.5
0.2
0
600
400

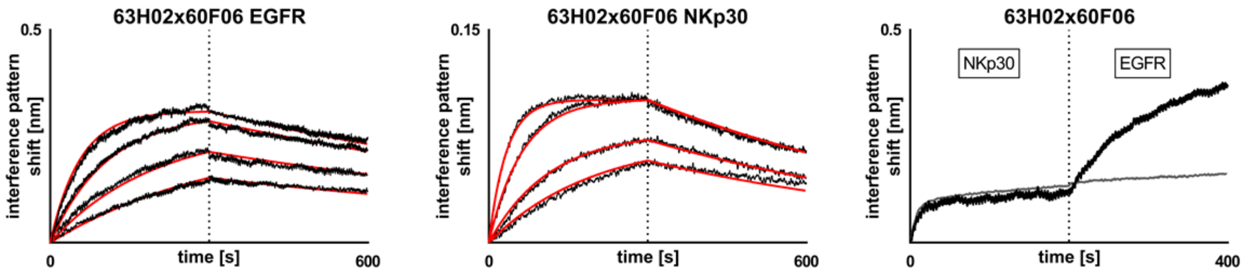

Supplementary Figure 6. Biochemical characterization of chimeric ultralong CDR-H3 common light chain bispecific antibodies via Biolayer interferometry. Kinetic measurements against recombinant human EGFR extracellular protein (left) or recombinant human NKp30 ECD (middle). Bispecific entities based on EGFR targeting paratope 60F06 were loaded onto sensor tips. After sensor rinsing, antigen binding was conducted at different concentrations for 300 s, followed by a dissociation step in kinetics buffer for 300 s. (Right) Simultaneous binding of bispecifics based on EGFR targeting paratope 60F06 against NKp30 ECD and EGFR ECD. Bispecifics were loaded to the sensor tips. After sensor rinsing two consecutive association steps were performed at 100 nM (Nkp30) or 100 nM (EGFR) for 200 s each.

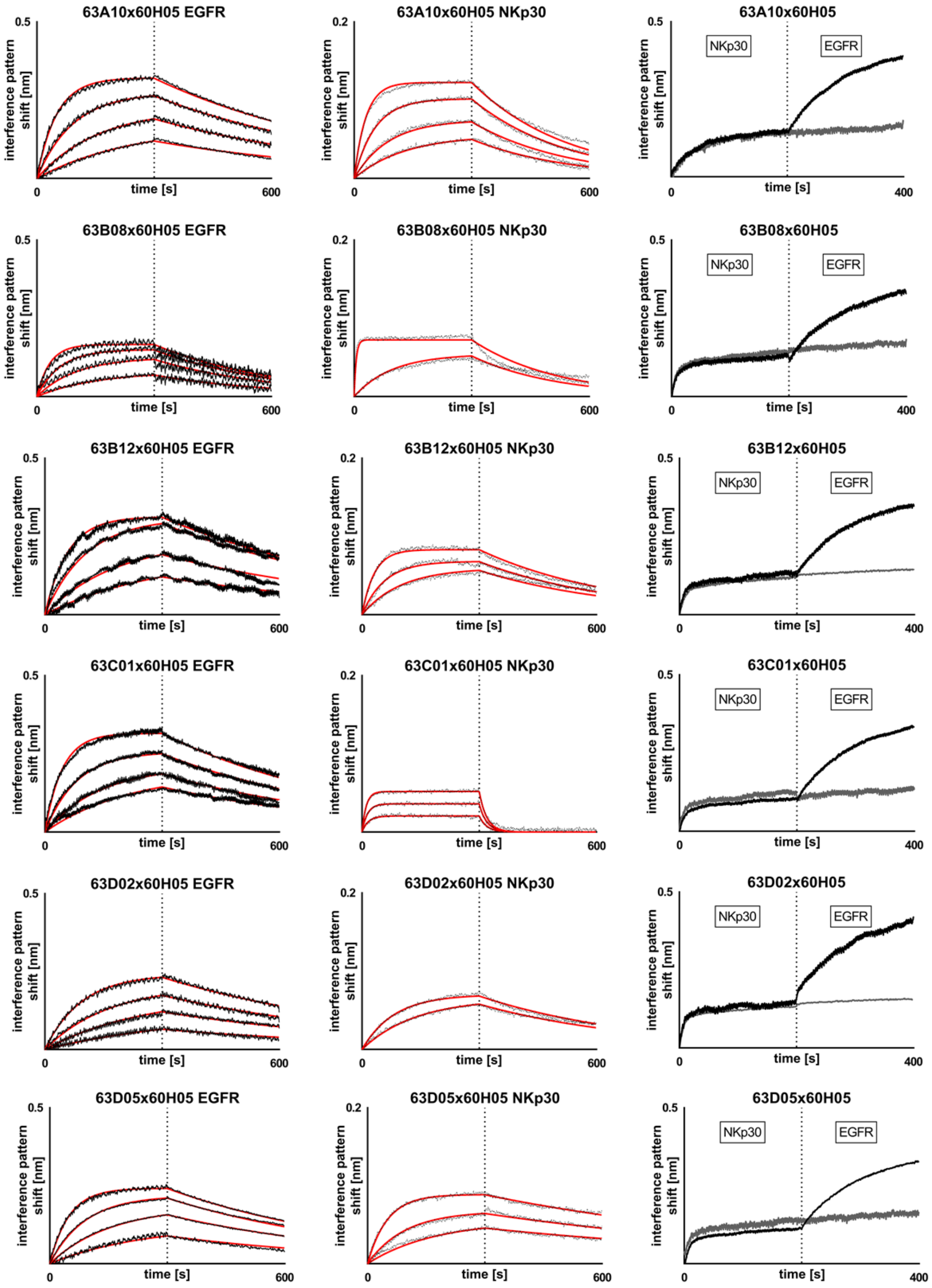
63A10x60H05 EGFR
63A10x60H05 NKp30
63A10x60H05
NKp30
EGFR
63B08x60H05 EGFR
63B08x60H05 NKp30
63B08x60H05
NKp30
EGFR
63B12x60H05 EGFR
63B12x60H05 NKp30
63B12x60H05
NKp30
EGFR
63C01x60H05 EGFR
63C01x60H05 NKp30
63C01x60H05
NKp30
EGFR
63D02x60H05 EGFR
63D02x60H05 NKp30
63D02x60H05
NKp30
EGFR
63D05x60H05 EGFR
63D05x60H05 NKp30
63D05x60H05
NKp30
EGFR
interference pattern shift [nm]
time [s]
0.5
0.2
0
600
400

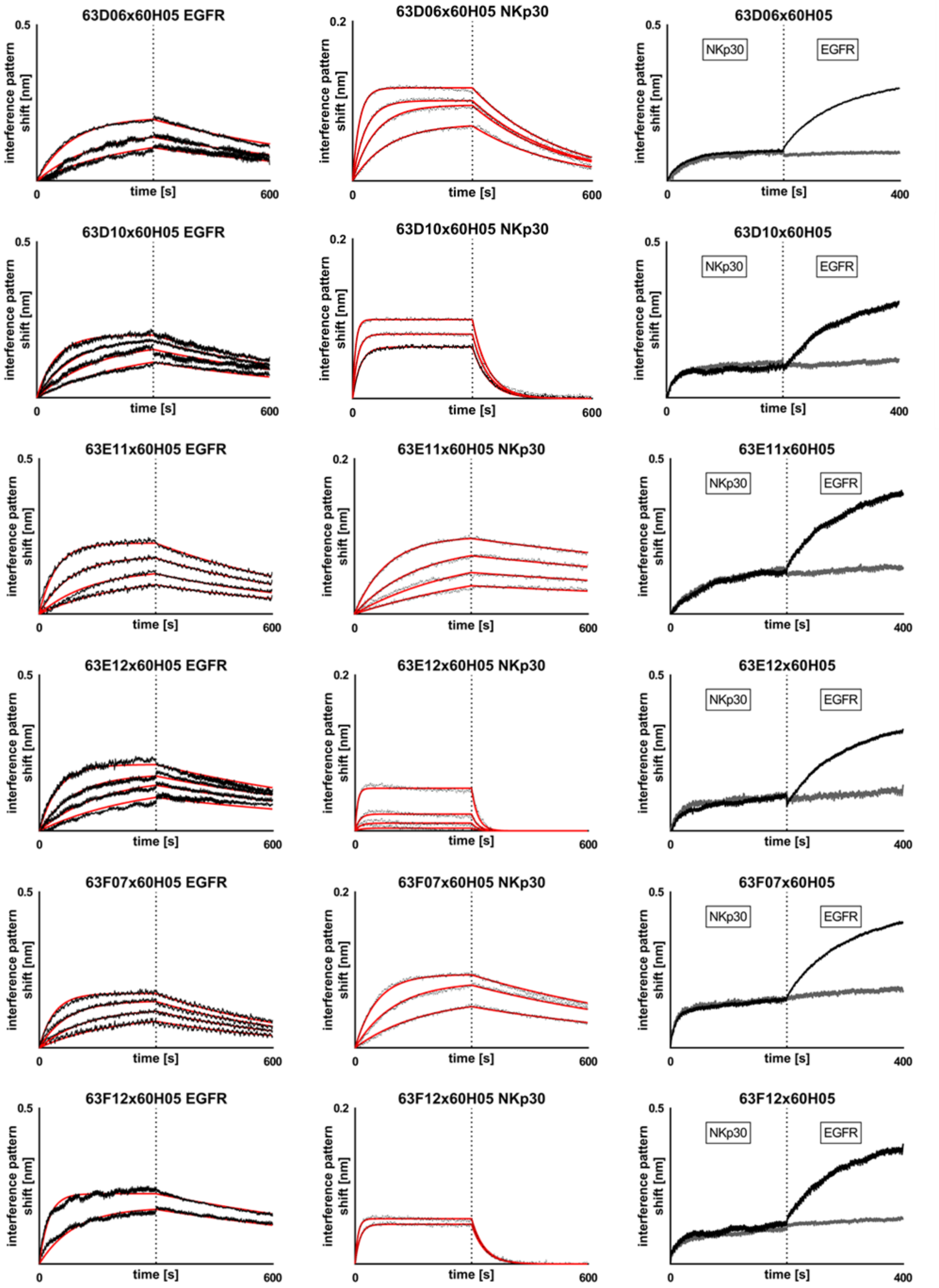
63D06x60H05 EGFR
63D06x60H05 NKp30
63D06x60H05
63D10x60H05 EGFR
63D10x60H05 NKp30
63D10x60H05
63E11x60H05 EGFR
63E11x60H05 NKp30
63E11x60H05
63E12x60H05 EGFR
63E12x60H05 NKp30
63E12x60H05
63F07x60H05 EGFR
63F07x60H05 NKp30
63F07x60H05
63F12x60H05 EGFR
63F12x60H05 NKp30
63F12x60H05
interference pattern shift [nm]
time [s]
0
600
400
0.5
0.2
NKp30
EGFR

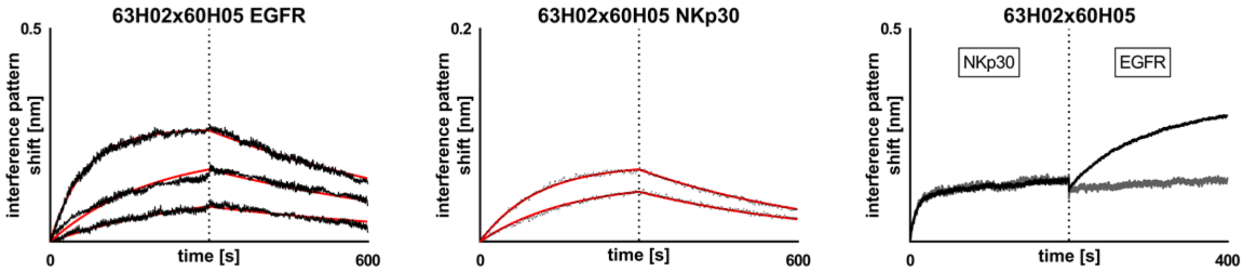

Supplementary Figure 7. Biochemical characterization of chimeric ultralong CDR-H3 common light chain bispecific antibodies via Biolayer interferometry. Kinetic measurements against recombinant human EGFR extracellular protein (left) or recombinant human NKp30 ECD (middle). Bispecific entities based on EGFR targeting paratope 60H05 were loaded onto sensor tips. After sensor rinsing, antigen binding was conducted at different concentrations for 300 s, followed by a dissociation step in kinetics buffer for 300 s. (Right) Simultaneous binding of bispecifics based on EGFR targeting paratope 60H05 against NKp30 ECD and EGFR ECD. Bispecifics were loaded to the sensor tips. After sensor rinsing two consecutive association steps were performed at 100 nM (Nkp30) or 100 nM (EGFR) for 200 s each.

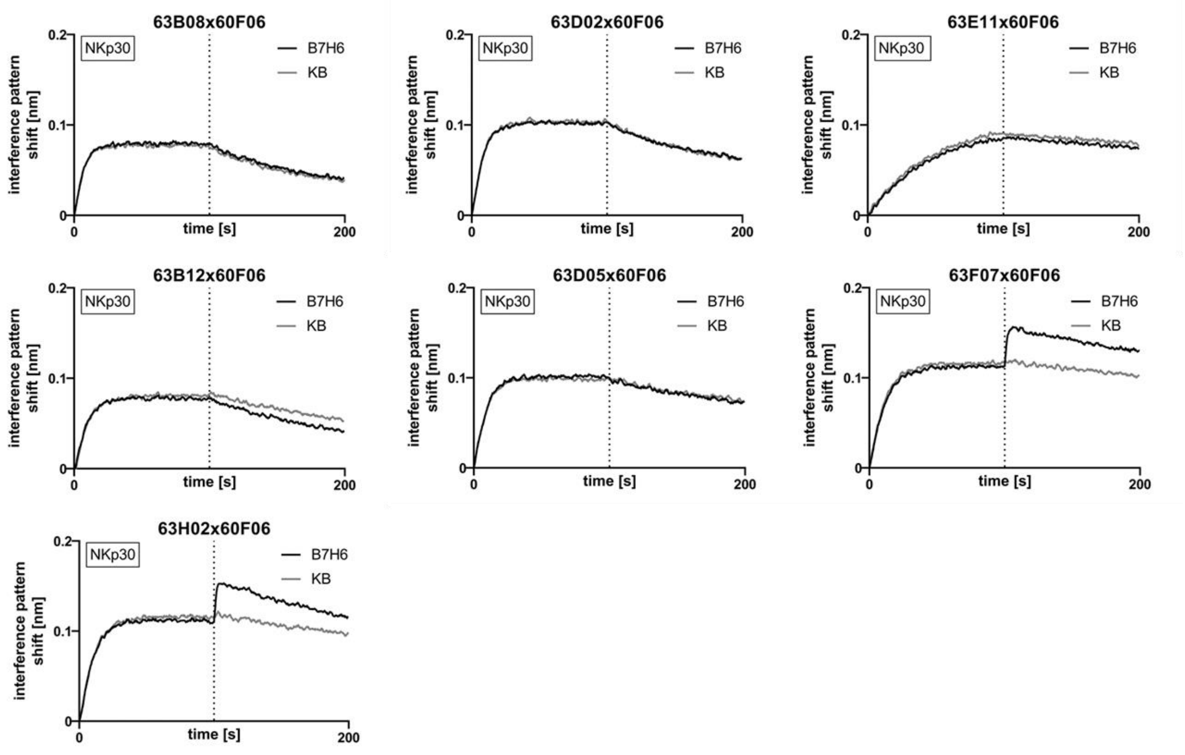

Supplementary Figure 8. Competition of bsAbs with B7-H6 for binding to NKp30 by Biolayer interferometry. Bispecific entities were loaded onto sensor tips. After sensor rinsing binding to 100 nM NKp30 was monitored for 100 s followed either by 100 s in KB (grey) or 1000 nM B7-H6 ECD.

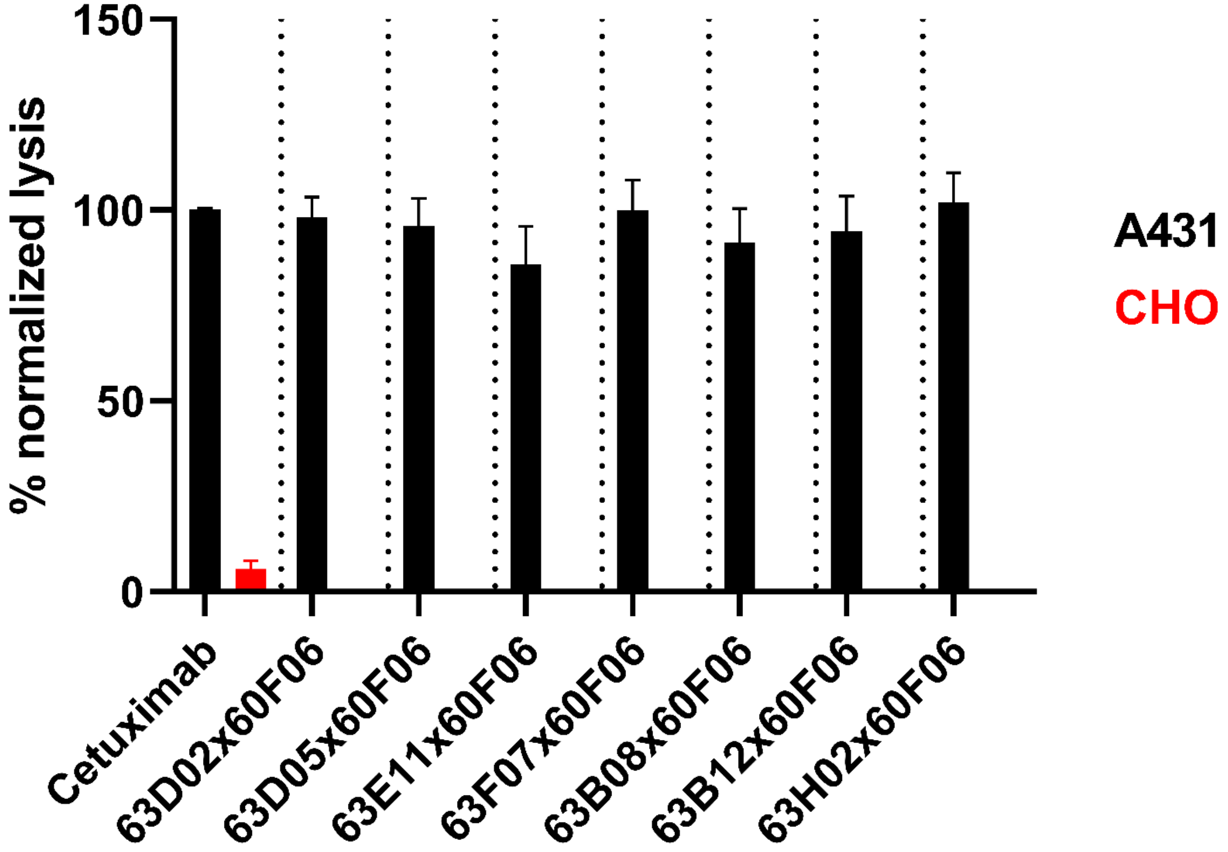

Supplementary Figure 9. Specificity of cytotoxic activity of generated chimeric ultralong CDR-H3 common light chain bsAbs. Fluorescence-microscopy based killing assay with EGFR-positive A431 (black) and EGFR-negative ExpiCHO™ (red) target cells as well as NK effector cells derived from PBMCs at an E:T ratio of 5:1. Analysis of maximum target cell killing (at a concentration of 50 nM for A431 and 500 nM for ExpiCHO™) via NK-mediated ADCC. Comparison of seven selected cow-derived bispecifcs. Cetuximab was included as control molecule. Data was normalized to allow for comparison of independent experiments. Graphs show normalized means ± SEM of n = 8 different healthy donors.

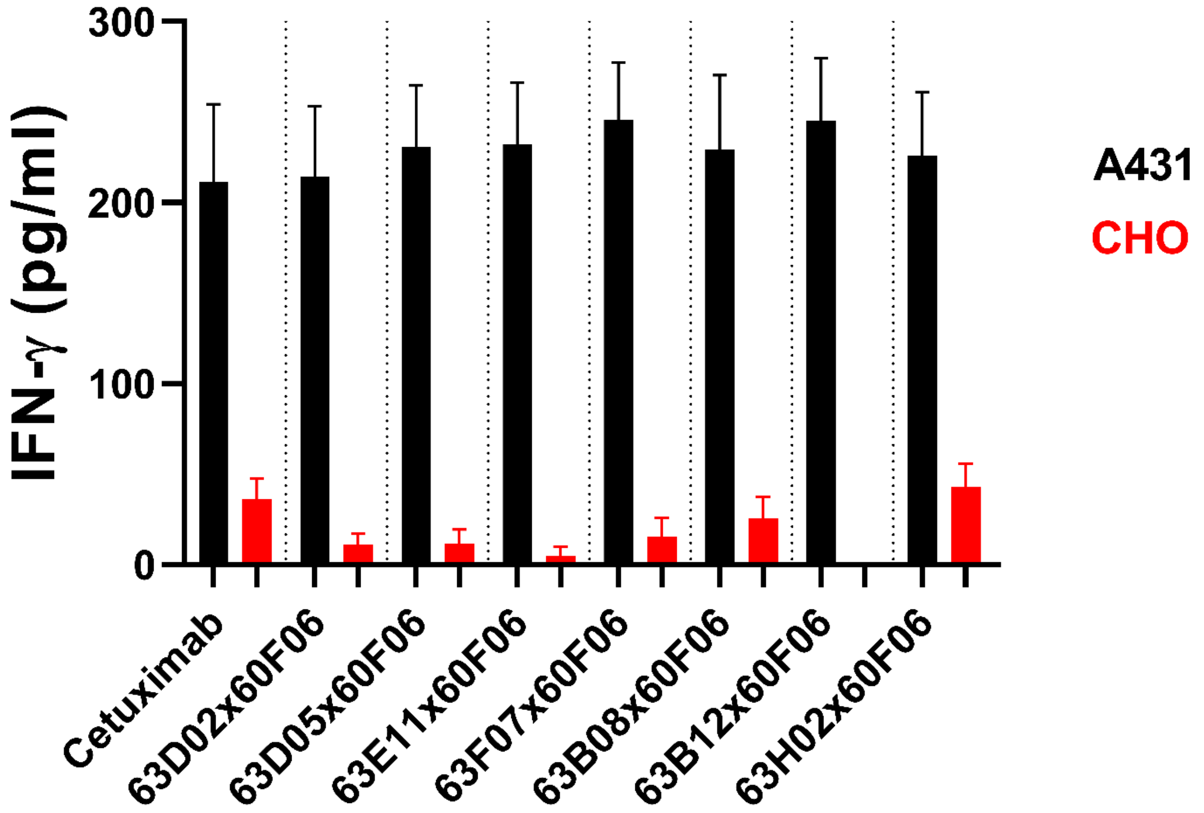

Supplementary Figure 10. Targeted NK cell mediated IFN-γ release triggered by cattle derived ultralong CDR-H3 bsAbs. Black: co-culture of EGFR positive A431 cells with PBMC isolated NK cells. Red: co-culture of EGFR negative ExpiCHO™ cells with PBMC isolated NK cells. Compounds were added at a concentration of 50 nM for A431 and 500 nM for ExpiCHO™. Cetuximab was included as control. For quantification cytokine HTRF kits were used. Purified NK cells were cocultured with A431 cells for 24 h at an E:T ratio of 5:1 prior to analysis. Graphs show normalized means ± SEM of n = 8 different healthy donors.

2. Supplementary Table

Supplementary Table 1. List of primers utilized for specific amplification of bovine ultralong CDR-H3 regions

Name	Sequence (5' to 3')
oVHBULL1_CDR3_GR_up	AGCAGCGTGACAACTGAGGACTCGGCCACATACTACTGTACTACTGTG
oVHBULL2_CDR3_GR_up	AGCAGCGTGACAACTGAGGACTCGGCCACATACTACTGTACTACTGTGCAC
oVHBULL4_CDR3_GR_up	AGCAGCGTGACAACTGAGGACTCGGCCACATACTACTGTACTACTGTGCACCAG
oVHBULL5_CDR3_GR_lo	GCCCTTGGTACTAGCTGAGGAGACGGTGACCAGGAGTCCTTGGCCCCA
oVHBULL6_CDR3_GR_lo	GCCCTTGGTACTAGCTGAGGAGACGGTGACCAGGAGTCCTTGGCCCCAGGCATC
oVL30_GR_up	CATTGCTGCTAAAGAAGAAGGGGTACAACTCGATAAAAGAGAAGCCGTTTTGAATCAACC
oVL30_GR_lo	GCGGGAACAGAGTGACCGAAGGGGCGGCCTTCGGCTGACCCAGAACGGTCAAAGTAGTAC
